# A Holistic Review of Oncological Drug Targets and Trajectories of Resistance in Cancer Therapy

**DOI:** 10.32604/or.2025.071209

**Published:** 2026-01-19

**Authors:** Harpreet Kaur, Dhrubalochan Rana, Sowvik Bag, Paramjeet Singh

**Affiliations:** 1 University Institute of Pharmaceutical Sciences, Panjab University, Chandigarh, 160014, India; 2 M. M. College of Pharmacy, Maharishi Markandeshwar (Deemed to be University), Mullana, Ambala, 133207, India; 3 Aurobindo Pharma, Durham, NC 27709, USA

**Keywords:** Targeted therapy, oncological drug targets, drug resistance, epidermal growth factor receptor, artificial intelligence

## Abstract

The prolonged and intricate history of oncological treatments has transitioned significantly since the introduction of chemotherapy. Substantial therapeutic benefits in cancer therapy have been achieved by the integration of conventional treatments with molecular biosciences and omics technologies. Human epidermal growth factor receptor, hormone receptors, and angiogenesis factors are among the established therapies in tumor reduction and managing side effects. Novel targeted therapies like KRAS G12C, Claudin-18 isoform 2 (CLDN18.2), Trophoblast cell-surface antigen 2 (TROP2), and epigenetic regulators emphasize their promise in advancing precision medicine. However, in many cases, the resistance mechanisms associated with these interventions render them ineffective in carrying out their functions. The purpose of this review is to provide a comprehensive and up-to-date examination of both established and emerging drug targets and mechanisms of treatment resistance in oncology. This review seeks to elucidate recent advancements, address persisting challenges, and explore opportunities for innovative developments in cancer target research. Additionally, it explores the growing role of artificial intelligence in reshaping cancer drug discovery and development frameworks as potential avenues for future research. In conclusion, innovative approaches in oncology, supported by pharmacological research, ongoing clinical trials, molecular biosciences, and artificial intelligence, are poised to significantly transform cancer treatment.

## 1 Introduction

Cancer, the most prevalent health concern in the modern era, has been among the extensively researched topics in the scientific community for many decades [1]. It is recognized as the second most significant causal factor of death, which continues to be the most serious health challenge around the world in over 60% of countries [2]. In 2020 alone, approximately 19.3 million new cases were estimated and diagnosed, and millions of people lost their lives to the disease [3]. It can be characterized by unrestrained proliferation of cells, vigorously invading their surrounding tissues and organs. Cancer can be divided into various categories depending on its histological origin, such as carcinomas, sarcomas, myeloma, lymphomas, leukemias, etc. Consequently, owing to their different cellular origins, a common therapeutic approach is ineffective in controlling their progression and management. Today, the oncological treatment options include surgery, radiation, chemotherapy, hormone therapy, targeted therapy, and immunotherapy, all of which are used either individually or in multimodality [4]. Several scientific and technological fields, like genomics, proteomics, bioinformatics, molecular biology, and epigenetics [5], cumulatively contributed to the evolution of anticancer therapies. However, a multitude of challenges [6] are associated with these approaches, ranging from heterogeneity of cancer, identifying the specific targets, tumor microenvironment, activation of alternate signaling pathways, poor prognosis, toxicity, and resistance to treatment. To overcome these challenges, targeted therapy [7] has emerged that precisely targets genetic mutations and pathways involved in cancer progression. Additionally, targeted therapy yields superior outcomes in conjunction with immunotherapy [8], chemotherapy, and radiotherapy [9] by overcoming drug resistance and suboptimal therapeutic response.

The targeted therapies function on the principle of identifying abnormal molecular markers, blocking signaling pathways, inhibiting angiogenesis, and promoting apoptosis. Although there have been tremendous advancements in identifying and targeting specific genes responsible for cancerous growth, multiple challenges are inevitable [10,11]. This led to an individually focused concept of personalized medicine that incorporates the amalgamation of technology, targeted therapy, and pharmacogenomics for diagnosis and treatment methods. Before targeted therapy, cancer treatments were limited to surgery, radiation, and chemotherapy. Chemotherapy involves systemic treatment encompassing infusion, injection, or solid form of anticancer drugs for curative and palliative objectives [12]. Chemotherapeutic drugs target oncological cells throughout the body, irrespective of their site of origin. This treatment method is effective in shrinking the cancerous cells; however, it also attacks the healthy cells in the patients. In most cases, radiation therapy follows chemotherapy and is localized in nature. Radiation therapy uses electromagnetic surges instead of anticancer drugs to eliminate cancerous cells from specific areas [9,13]. The side effects of chemotherapy and radiation therapy range from nausea, vomiting, hair loss, anemia, tiredness, etc. These therapies target cancer cells by inflicting genotoxic stress in cancer cells, inhibiting replication, and inducing apoptosis [12,13]. However, the challenges and resistance mechanisms linked to these therapies have facilitated the advancement of precision medicine and targeted therapy over the years. Promise in patient care can be ascertained through optimization of treatment using targeted therapy. Enzyme-specific inhibitors, mitogenic receptors, and intracellular signaling proteins are usually tailored in precision medicine to target biological processes within cancer cells [14]. Improvement in target selection is extremely tedious and a huge hurdle in the development of targeted therapies. The U.S. Food and Drug Administration (FDA) plays a vital role in the drug approval process and regulatory frameworks by updating and validating therapeutic targets. The FDA-approved anticancer medications that fall under the ATC categories L01 (Antineoplastic agents) and L02 (Endocrine therapy) can be generally categorized into three functional groups, depending on their mechanism of action and therapeutic strategies: chemotherapy, targeted therapy, and hormonal therapy [15]. The FDA’s Center for Drug Evaluation and Research (CDER) has approved around 36 new oncology drugs (including new molecular entities and biologics) in 2025 to date [16], some of which are indicated in Table 1. Thus, the FDA plays a crucial role in cancer care and is responsible for carrying out the approval, regulation, and monitoring of current and newly evolving drugs and biologics used in treatment. The FDA not only ensures post-market surveillance of acknowledged oncological drug targets but also tracks and controls new drug development. The well-established oncological drug targets constitute kinases (EGFR, BRAF/MEK pathway, HER2) [17], hormone receptors (ER and AR) [18], angiogenesis factor (VEGF) [19], and DNA-interacting targets (topoisomerase and PARP) [20]. Several novel therapeutic drugs are paving the way for effective cancer regimens by targeting multiple signaling pathways and mechanisms highlighted in Table 1.

**Table 1:** Regulatory approval status and mechanistic pathways of some novel oncological drugs in different therapeutic areas

S. No.	Generic name	Trade name	Date of approval	Drug class	Mechanism of action	Pathway	Trial identifier	Therapeutic area	Status
1.	Cemiplimab-rwlc	Libtayo	10/08/2025	Immune checkpoint inhibitor (PD-1)	Monoclonal antibody targeting PD-1 receptor, blocking PD-1/PD-L1 interaction	PD-1/PD-L1 pathway	NCT03969004	Cutaneous squamous cell carcinoma (CSCC)	Approved
2.	Lurbinectedin + Atezolizumab	Zepzelca, Tecentriq, Tecentriq Hybreza	10/02/2025	Lurbinectedin: DNA minor groove binder and Atezolizumab: Immune checkpoint inhibitor (PD-L1)	Lurbinectedin: binds DNA and inhibits transcription, Atezolizumab: monoclonal antibody targeting PD-L1, blocking PD-1/PD-L1 interaction	DNA transcription inhibition and PD-1/PD-L1 pathway	NCT05091567	Extensive-stage small cell lung cancer (ES-SCLC)	Approved
3.	Imlunestrant	Inluriyo	9/25/2025	Estrogen receptor antagonist	Binds to and antagonizes the estrogen receptor (ER), including ESR1-mutated forms, inhibiting ER-mediated transcription	ER signaling pathway	NCT04975308	ER-positive, HER2-negative advanced/metastatic breast cancer	Approved
4.	Pembrolizumab and berahyaluronidase alfa-pmph	Keytruda Qlex	9/19/2025	Immune checkpoint inhibitor (PD-1)	Monoclonal antibody targeting PD-1 receptor, blocking PD-1/PD-L1 interaction	PD-1/PD-L1 pathway	NCT05722015	Metastatic non-small cell lung cancer (NSCLC)	Approved
5.	Selumetinib	KOSELUGO	9/10/2025	MEK inhibitor	Selectively inhibits MEK1/2, blocking the RAS-RAF-MEK-ERK signaling pathway	RAS-RAF-MEK-ERK pathway	SPRINT Phase II Stratum I study and the SPRINKLE study	Pediatric neurofibromatosis type 1 (NF1) with symptomatic, inoperable plexiform neurofibromas	Approved
6.	Gemcitabine intravesical system	Inlexzo	9/9/2025	Antimetabolite /Cytotoxic	Nucleoside analog incorporated into DNA, inhibiting DNA synthesis and inducing apoptosis	DNA synthesis inhibition	NCT04640623	BCG-unresponsive non-muscle invasive bladder cancer (NMIBC) with CIS with/without papillary tumors	Approved
7.	Zongertinib	Hernexeos	8/8/2025	Kinase inhibitor	Selectively inhibits HER2 (ERBB2) tyrosine kinase domain (TKD) activating mutations, blocking downstream signaling and tumor growth	HER2/ERBB2 signaling pathway	NCT04886804	Unresectable or metastatic non-squamous NSCLC with HER2 TKD mutations	Accelerated Approval
8.	Dordaviprone	Modeyso	8/6/2025	Protease activator	Activates specific proteases, leading to antitumor activity in H3 K27M-mutant diffuse midline glioma	Protease activation	ONC006 (NCT02525692), ONC013 (NCT03295396), ONC014 (NCT03416530), ONC016 (NCT05392374), ONC018 (NCT03134131)	H3 K27M-mutant diffuse midline glioma (adult and pediatric)	Accelerated approval granted
9.	Sunvozertinib	Zegfrovy	7/2/2025	Tyrosine Kinase Inhibitor (TKI)	Selectively inhibits mutant EGFR, especially exon 20 insertion variants	EGFR Signaling Pathway	NCT03974022	Non-Small Cell Lung Cancer (NSCLC)	Accelerated approval granted
10.	Linvoseltamab-gcpt	Lynozyfic	7/2/2025	Bispecific T-cell engager antibody (BiTE)	Engages CD3^+^ T cells and BCMA+ myeloma cells to promote T-cell–mediated cytotoxicity	Immune synapse formation/T-cell activation pathway	NCT03761108	Hematologic Malignancy (Multiple Myeloma)	Accelerated approval granted
11.	Datopotamab deruxtecan-dlnk	Datroway	6/23/2025	Antibody–Drug Conjugate (ADC)	Antibody targets TROP2 and delivers a topoisomerase	TROP2-mediated internalization and cytotoxic drug release	TROPION-Lung05 (NCT04484142), TROPION-Lung01 (NCT04656652)	Non-Small Cell Lung Cancer (NSCLC)	Accelerated approval granted
12.	Tafasitamab-cxix	Monjuvi	6/18/2025	Monoclonal antibody (Fc-engineered anti-CD19)	Targets CD19 on B-cells, enhancing antibody-dependent cellular cytotoxicity (ADCC)	B-cell receptor (BCR) signaling pathway	NCT04680052	Hematologic Malignancy (Follicular Lymphoma)	Approved
13.	Pembrolizumab	Keytruda	6/12/2025	Immune checkpoint inhibitor	Anti–PD-1 monoclonal antibody—blocks PD-1 to enhance T-cell–mediated anti-tumor response	PD-1/PD-L1 immune checkpoint pathway	KEYNOTE-689 (NCT03765918)	Head and Neck Cancer	Approved
14.	Mitomycin	Zusduri	6/12/2025	Alkylating agent/DNA crosslinker	Induces DNA crosslinking, inhibiting DNA synthesis and triggering cancer cell death	DNA damage response and apoptosis pathways	ENVISION (NCT05243550)	Urologic Cancers	Approved
15.	Taletrectinib	Ibtrozi	6/11/2025	Kinase inhibitor (ROS1 and NTRK fusion inhibitor)	Inhibits ROS1 tyrosine kinase activity, blocking downstream signaling pathways critical for tumor growth	Tyrosine kinase signaling pathways—including MAPK/ERK and PI3K/AKT	TRUST-I (NCT04395677) and TRUST-II (NCT04919811)	Non-Small Cell Lung Cancer (NSCLC)	Approved
16.	Darolutamide	Nubeqa	6/3/2025	Androgen receptor (AR) antagonist	Blocks androgen receptor signaling, inhibiting prostate cancer cell growth	Androgen receptor signaling pathway	ARANOTE (NCT02799602)	Prostate Cancer	Approved
17.	Retifanlimab-dlwr	Zynyz	5/15/2025	Immune checkpoint inhibitor (anti-PD-1 monoclonal antibody	Blocks PD-1 receptor, enhancing T-cell-mediated immune response against tumor cells	PD-1/PD-L1 immune checkpoint pathway	POD1UM-303/Inter AACT 2 (NCT04472429)	Squamous Cell Carcinoma of the Anal Canal (SCAC)	Approved
18.	Telisotuzumab vedotin-tllv	Emrelis	5/14/2025	Antibody–Drug Conjugate (ADC) targeting c-Met with microtubule inhibitor payload	Binds c-Met receptor and delivers cytotoxic agent, disrupting microtubules, causing cancer cell death	c-Met receptor signaling pathway	LUMINOSITY study (NCT03539536),	Non-Small Cell Lung Cancer (NSCLC)	Accelerated approval granted

Recent advancements in technology have facilitated researchers in investigating emerging targets such as Kirsten Rat Sarcoma viral oncogene homolog (KRAS) [21], Claudin-18 isoform 2 (CLDN18.2) [22], Trophoblast cell-surface antigen 2 (TROP2) [23], Lymphocyte-activation gene 3 (LAG-3), T-cell immunoglobulin and mucin-domain containing-3 (TIM-3) [24]. In comparison to traditional therapies, these mechanistic pathways exhibit significantly enhanced efficacy and reduced toxicity. Their capacity to target a broad spectrum of malignancies positions them as highly effective options for oncological treatments [23]. The scope of this review is to provide an exploration of established and emerging drug targets while also addressing mechanisms of resistance, with insights into future directions for drug development in oncology.

## 2 Established Drug Targets

The standard cancer treatment regimens employ several therapies in singularity or in combination for cancer management. The established oncological drug targets can be characterized as receptors and non-receptors. The receptors offer binding sites for small molecules, antibody-drug conjugates, monoclonal antibodies, etc, leading to inhibition of their expression, impeding tumor growth, and stalling the progression of disease.

### 2.1 Human Epidermal Growth Factor Receptor

**Figure 1:**
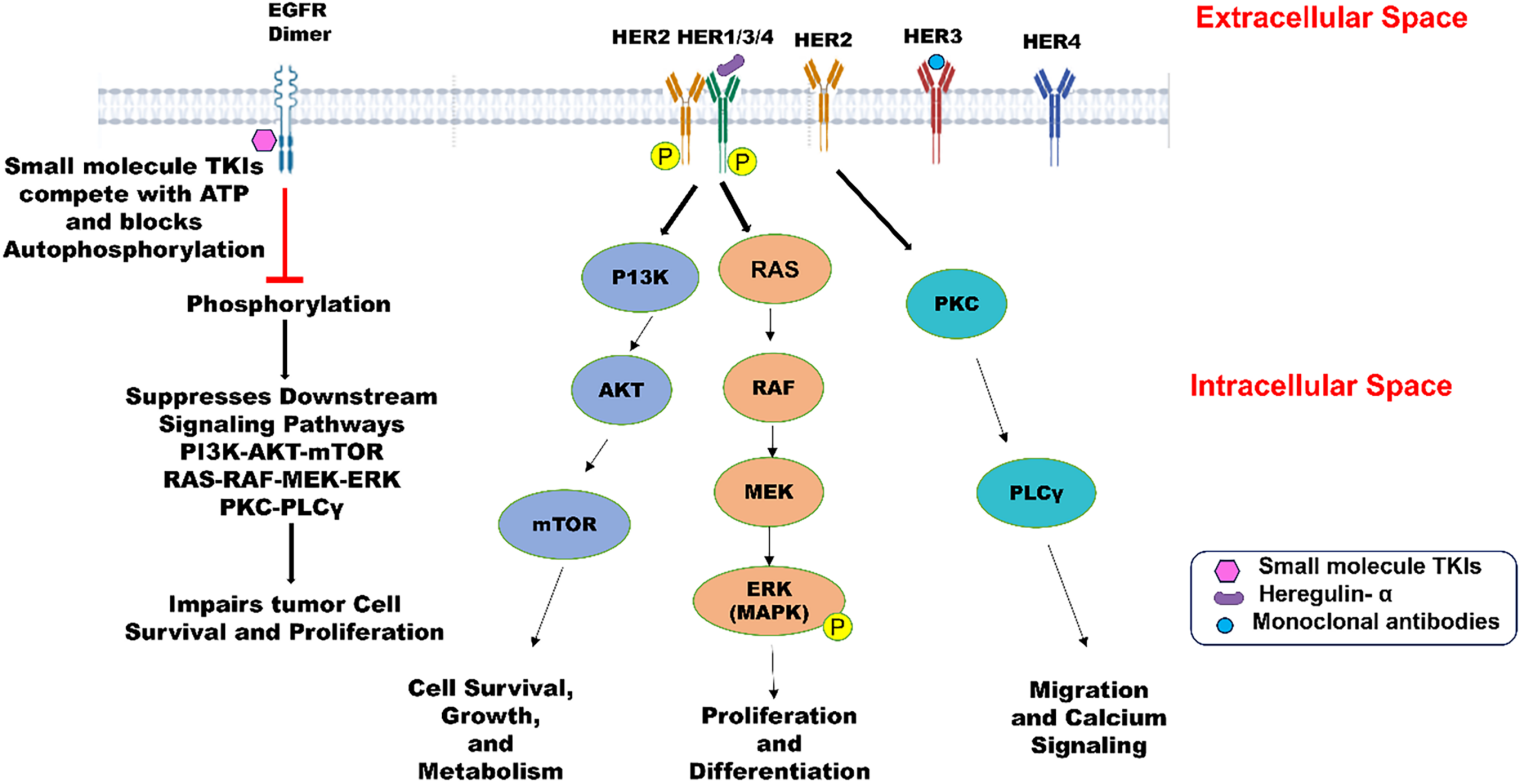
The structure of human epidermal growth factor receptor (HER1/EGFR, HER2, HER3, HER4) comprises an extracellular domain, transmembrane domain, and intracellular kinase domain (except HER3). Epidermal growth factors like EGF, TGF, Heregulin-α are the ligands that bind to EGFR and trigger cellular responses. HER2-HER3 dimers lead to phosphorylation of tyrosine kinase residues in the cytoplasm and promote PI3K → AKT → mTOR pathway, leading to cell survival, growth, and metabolism. HER2-HER3 heterodimerization can also activate the MAPK pathway involved in proliferation, differentiation, and cell survival. HER2 activation initiates PKC → PLCγ, promoting migration and calcium signaling. Small molecules like TKIs bind to the intracellular kinase domain of tyrosine kinase receptors and block the ATP-binding site, preventing phosphorylation and downstream signaling. Created with BioRender.com. Information adapted from References [29,31]

The human epidermal growth factor receptor (HER/ErbB) family is a classic example of an oncological drug target in cancer treatment. This family comprises four transmembrane tyrosine kinase receptors [25], namely ErbB1/HER1 or epidermal growth factor receptor (EGFR), ErbB2/HER2, ErbB3/HER3, and ErbB4/HER4. All these receptors are normally expressed in cells for differentiation, cell growth, tissue repair, and embryonic development by activating intracellular signaling pathways, including PI3K/AKT, MAPK, PKC → PLCγ, RAS-MAPK, and JAK/STAT. However, their overexpression or mutation leads to uncontrolled signaling and tumor growth, leading to breast cancer [26], as illustrated in Fig. 1. The three receptors, except ErbB4/HER4, are significant in targeted therapy for treating different types of cancers. Non-small cell lung cancer (NSCLC), head and neck squamous cell carcinoma (HNSCC), and colorectal cancer (CRC) resulting from overexpression of ErbB1/HER1 can be managed successfully by treating with drugs like erlotinib, gefitinib, and cetuximab. Erlotinib and gefitinib, also classified as reversible inhibitors, are small-molecule tyrosine kinase inhibitors [27]. These inhibitors bind to the ATP-binding site of the intracellular EGFR tyrosine kinase domain, blocking receptor autophosphorylation and starting a cascade of complex cell biochemistry known as downstream signaling (Fig. 1). Gefitinib arrests the cell cycle at the G1 phase by blocking the mitogen-activated protein kinase pathway (MAPK) and P13K/AKT pathways involved in apoptosis and cell survival. Gefitinib was also reported to be more tolerable and cost-effective as compared to erlotinib. Cetuximab, a reversible extracellular EGFR inhibitor, blocks the binding site of growth factors, inhibiting receptor activation, dimerization, and downstream signaling [28]. Breast cancer and stomach adenocarcinoma exhibit aggressive tumor growth due to overexpression of ErbB2/HER2 tyrosine kinase receptors [29]. Unlike other family members, HER2 does not need a ligand for activation. On the contrary, it dimerizes with other ligand-bound HER receptors, activating downstream pathways like PI3K/AKT and MAPK/ERK. Trastuzumab (Herceptin), a monoclonal antibody (IgG1) was approved by the FDA in 1998 for targeting HER2 receptors in HER2-positive metastatic breast cancer. Additionally, Trastuzumab is used synergistically with Pertuzumab effectively blocking HER2 signaling and yielding better results [29]. HER3 exhibits relatively low expression in tumors due to a lack of intrinsic kinase activity. This necessitates its dimerization with another HER receptor to initiate downstream signaling. This dimerization leads to activating the PI3K/AKT signaling pathway, promoting tumor growth and resistance against cancer therapies [30]. There are several HER3-targeted agents under clinical development. Out of several anti-HER3 monoclonal antibodies tested for therapeutic use in cancer treatment, three molecules demonstrated therapeutic potential and advanced to phase II and III clinical trials [31]. Daiichi Sankyo Co., Ltd. sponsored a potential monoclonal antibody, Patritumab (U3-1287), which is under phase III clinical trial [30]. This targeted therapy selectively targets the HER3 receptor, rendering its extracellular domain unavailable for binding with other receptors. It leads to inhibition of dysregulated cellular pathways leading to cancer progression. In the same direction, MorphoSys/Novartis and GlaxoSmithKline are conducting research on Elgemtumab (LJM716) and GSK2849330, respectively [30], within the framework of phase I clinical trials. An antibody drug conjugate, Patritumab deruxtecan (U3 1402), is also undergoing phase I/II clinical trials. This drug comprises a conjugate of a human monoclonal antibody and a topoisomerase 1 inhibitor, which, upon release inside a cancer cell, leads to apoptosis. HER3-directed agents are relatively new in oncological research and are gaining momentum with time as potential therapeutic agents.

### 2.2 Hormone Receptors

The steroid hormone receptors (SHRs) perform their intracellular activities through their ligand-dependent intracellular transcription factors. SHRs mediate intercellular signaling from a steroid/hormone to the transcriptional targets by interacting with specific DNA motifs in the genome and various coregulatory proteins that consist of activators/corepressors [32]. Any perturbation in this interaction may result in the development of malignancies owing to altered gene expression. The transcriptomics studies on steroid hormone receptors facilitated novel steroid-based targeted therapies for cancer. The hormone-activated transcription factors comprise nuclear hormone receptors for glucocorticoid (GR), estrogen (ER), progesterone (PR), androgen (AR), and mineralocorticoid (MR). Significant examples of SHRs include the estrogen receptor (ER) in breast cancer [33] and the androgen receptor (AR) in prostate cancer. The estrogen receptor (ER) is classified into two types: Erα, derived from the actual estrogen receptor protein extracted from the uterus of rats, and Erβ cloned from a rat prostate cDNA library. Among these, Erα is the predominant estrogen receptor expressed in breast cancer, while the role of Erβ remains ambiguous as it is not yet an established standard clinical biomarker. Hormone-sensitive cancers can be managed with anti-estrogen drugs or aromatase inhibitors. The binding between estrogen and ERα in the intracellular domain leads to the release of HSP90, HSP70, and cyclophilin [34]. ERα dimerizes and binds to estrogen response elements (EREs) on DNA, leading to transcription of responsive genes like TFF1. The TFF1 protein secretion stimulates tumor growth in cancer. The binding of anti-estrogen medications, such as tamoxifen, to estrogen receptors renders them nonfunctional, while aromatase inhibitors work by decreasing overall estrogen levels. Drugs like figitumumab and lapatinib affect the transcriptional activity of the estrogen-related receptor alpha while targeting the insulin-like growth factor (IGF) and epidermal growth factor (EGF) pathways [34]. This mechanism leads to programmed cell death in oncological targets. Further transcriptomic studies can effectively predict resistance to figitumumab and lapatinib. Hence, omics can play an important role in drug development in these drug categories.

*Androgen Receptor (AR) in Prostate Cancer*

Metastatic prostate cancer is treated with androgen receptor antagonists, which may be steroidal or non-steroidal in nature. However, in many cases, the disease progresses to a more aggressive form called castration-resistant prostate cancer, driven by elevated expression of the androgen receptor. In the normal prostate, AR is expressed primarily in androgen receptor-positive (AR^+^) luminal cells as well as mesenchymal cells, with only low expression in AR-negative basal epithelial cells. Various studies conducted with animal models [35] also indicates that in healthy prostate epithelium, the androgen receptor (AR) independently regulates cell proliferation and promotes the differentiation of epithelial cells. Therefore, AR plays a significant role as a tumor suppressor. AR, a transcriptional regulator of cell proliferation, has four regions: from the N-terminal, an NH_2_-terminal transactivation domain (NTD), a DNA-binding domain (DBD), a hinge region, and a ligand-binding domain (LBD). Shorter glutamine repeats present in NTDs are associated with high transcriptional activity of AR, leading to a higher risk of prostate cancer. Androgen receptor amplification in the AR gene, located on chromosome X (Xq11-12), is prevalent in about half of the population of castration-resistant prostate cancer individuals. Anti-androgens such as bicalutamide, flutamide, and enzalutamide have a greater affinity for the ligand-binding domain of AR [36]. However, the tumors eventually become resistant to the drugs by expressing higher levels of the AR. It necessitates qualitative work on next-generation anti-androgens showing high binding affinity for AR. The second-generation androgen receptor antagonists, enzalutamide and apalutamide (ARN-509) demonstrate significant results while also exhibiting a comparable profile of side effects. However, apalutamide (ARN-509) came forth as more promising than enzalutamide. Instead of agonistic activity, apalutamide inhibits the nuclear localization and DNA binding of AR in prostate cancer cells [37]. This medication offers a measure of metastasis-free survival in patients for up to two years. Several clinical trials [38] are evaluating whether the combination of anti-androgens like enzalutamide, apalutamide, or abiraterone provides superior outcomes in non-metastatic CRPC.

**Figure 2:**
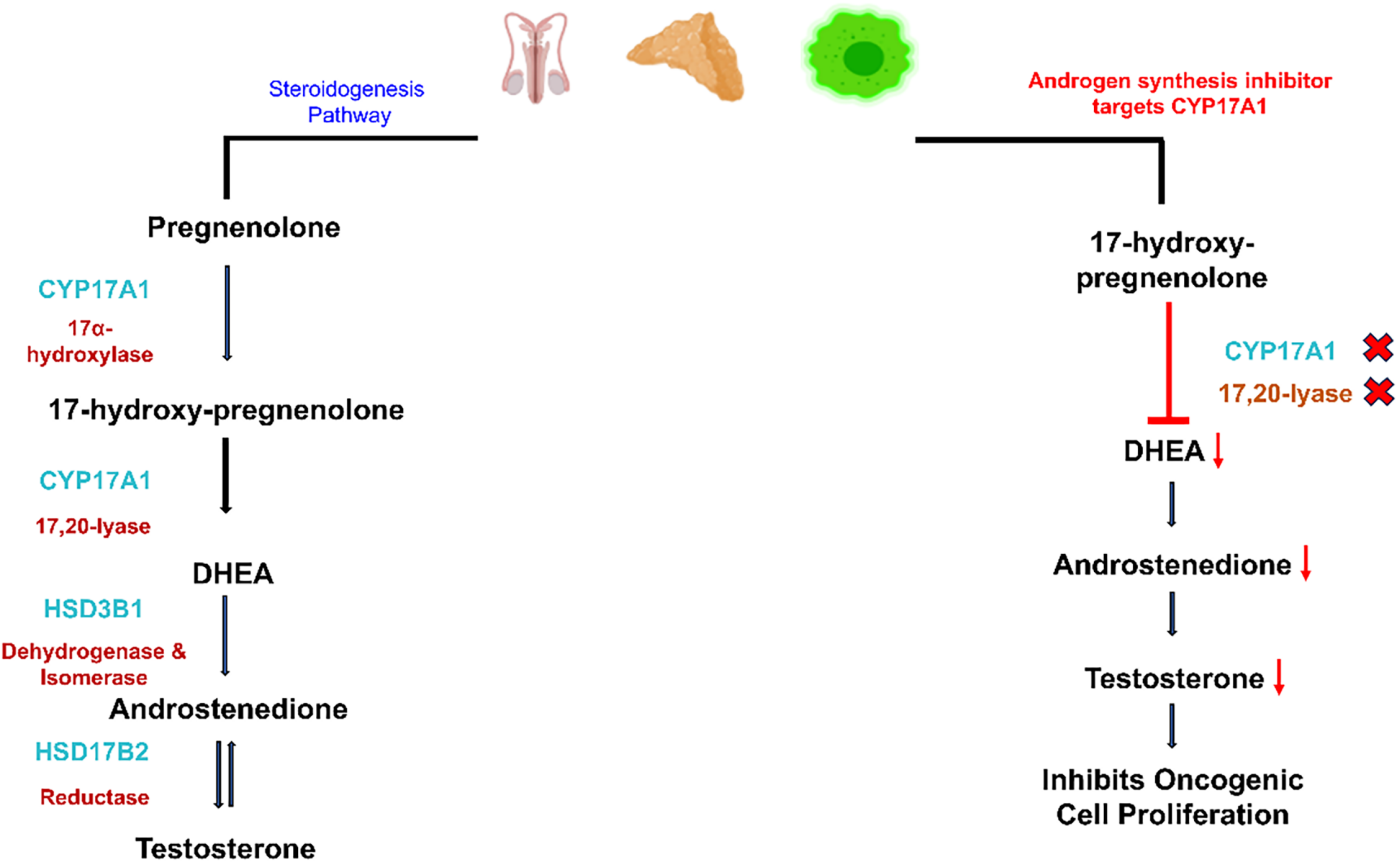
The Hypothalamus, the body’s control center, directs the anterior pituitary gland (adenohypophysis) to release LH in the blood, traveling to Leydig cells and stimulating them to release testosterone. The activation of the steroidogenesis pathway leads to the conversion of cholesterol into pregnenolone, which further changes to 17α-Hydroxypregnenolone by 17α-hydroxylase activity performed by CYP17 (Cytochrome P450 17A1). CYP17 enzyme adds a hydroxyl group (–OH) at the 17α position of pregnenolone and progesterone. The subsequent step incorporates the same enzyme for performing 17,20-lyase activity, releasing DHEA through the cleavage of the side chain located between carbons 17 and 20. Dehydrogenase and isomerase, and reductase activities are carried out by HSD3B1 and HSD17B2 enzymes, respectively, to release androstenedione and testosterone. However, androgen synthesis inhibitors (ASIs) as drug targets block the CYP17A1 enzyme, leading to inhibition of 17α-hydroxylase and 17,20-lyase activities. Resultantly, the concentration of DHEA and androstenedione drops, leading to low testosterone levels in the blood. The decline in testosterone levels leads to inactivity of androgen receptors. No transcription of cell cycle regulators and other proliferative genes inhibits oncological expression. In the figure, X represents inhibition or blockage, and ↓ represents a drop in concentration. Created with BioRender.com. Information adapted from References [39]

Therapies aiming to inhibit AR signaling, called AR signaling inhibitors (ARSIs), have been in clinical use for more than a decade, but the effectiveness of these measures is limited in duration, and the majority of patients develop castration-resistant prostate cancer. FDA-approved androgen synthesis inhibitors like Abiraterone acetate [39] inhibit the production of androgen in the testes, adrenal glands, and oncological cells by blocking the enzyme, cytochrome P450 17 alpha-hydroxylase (CYP17), as highlighted in Fig. 2.

### 2.3 Angiogenesis Factors

**Figure 3:**
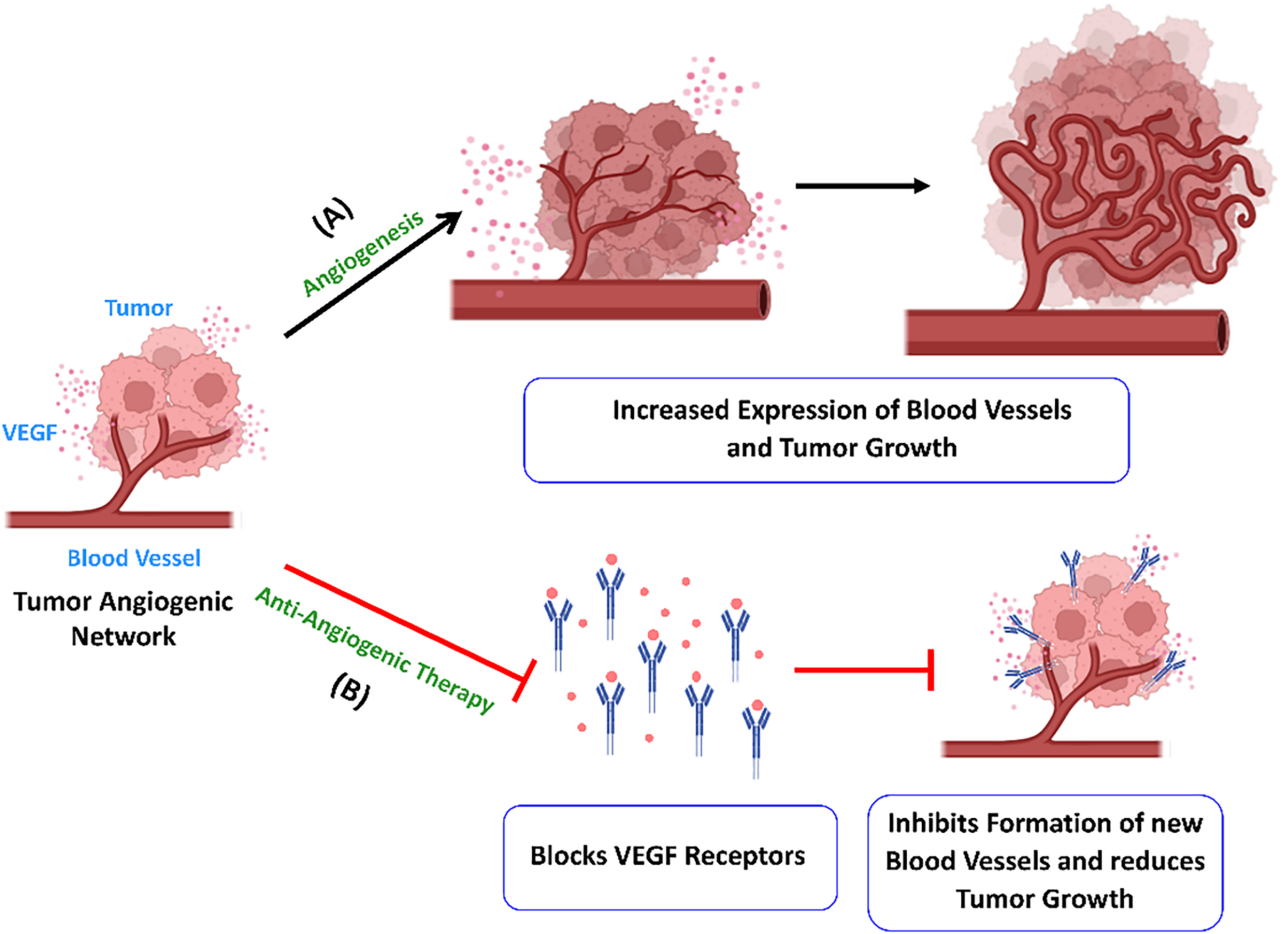
Angiogenesis enhances the growth of tumor cells. (**A**) The tumor cells secrete VEGF, which leads to the formation of new blood vessels and tumor growth. (**B**) Anti-angiogenic medications like bevacizumab target VEGF-A and prevent activation of the receptor. It inhibits new blood vessel formation in tumors and limits their growth and metastasis. Created with BioRender.com. Information adapted from References [40,41]

Angiogenesis is the process of blood vessel formation, from pre-existing ones, for maintaining normal body functions like embryogenesis, angioblast differentiation, wound healing, and blood vessel growth during the female reproductive cycle. Nonetheless, this same mechanism can facilitate tumor advancement and the metastasis of cancer. This process is regulated by a multitude of pro-angiogenic and angio-inhibitory signaling molecules [40]. Angiogenesis includes the endothelial cell activation and remodeling, vascular tubulogenesis, and the construction of vascular networks. High vascularization in cancer tumors, as compared to healthy cells, results from imbalanced expression of angiogenic factors. The formation of abnormal vascular networks is related to the unique inflammatory, metabolically stressed microenvironment within tumors. Vascular endothelial growth factor (VEGF) is regarded as a key angiogenesis factor. It is a signaling molecule that promotes endothelial cell proliferation, migration, and new capillary formation. However, apart from numerous non-endothelial cells, it is also expressed in tumor cells [41]. A therapeutic agent that targets the pro-angiogenic activity of vascular endothelial growth factor (VEGF) inhibits neovascularization, thereby presenting a potential anti-VEGF strategy for the treatment of cancers. The US Food and Drug Administration (FDA) has approved several anti-angiogenic drugs (AADs) that target the VEGF pathway for cancer treatment. Bevacizumab, Ramucirumab, Sorafenib (Nexavar), and Cabozantinib (Cabometyx) are some monospecific antibodies and orally active small molecules that target VEGF and receptor tyrosine kinases like TIE2, c-MET, PDGFRs, and RET [42,43]. These anti-angiogenic drugs act through complex mechanisms, including anti-angiogenesis (Fig. 3), vascular normalization, angiogenic regression, vascular barrier restoration, and the alteration of immune functions. Monospecific drugs such as Bevacizumab and fluorouracil-based combination chemotherapy [43] have demonstrated significant clinical improvement in survival in metastatic colorectal cancer patients. It neutralizes only one angiogenic factor, VEGF-A, by blocking its interaction with VEGF receptors. Eli Lily funded research on Ramucirumab for hepatocellular carcinoma and increased α-fetoprotein concentrations after Sorafenib therapy was established as the first successful phase 3 study in a biomarker-selected patient population [42]. Recently, first-line non-sorafenib regimens for advanced HCC have also shown promising results in clinical outcomes [44]. However, the study was limited by its small and highly selected patient population, as well as its single-arm design, which precluded direct comparisons with other therapies. Future studies should include larger, randomized cohorts with more diverse liver function status to allow robust comparison across prior therapy types.

Soluble receptors such as aflibercept can neutralize two or three VEGFR1 ligands, while tyrosine kinase inhibitors have multiple targets like VEGFR-2/3, PDGFR-β, RAF, FLT3, KIT, and RET. Due to their diverse range of targets, tyrosine kinase inhibitors (TKIs) frequently present significant toxicity profiles, limiting their long-term clinical application [17,19]. This hinders their opportunity to serve as a combination therapy with other anti-cancer agents.

### 2.4 DNA-Interacting Targets

DNA-interacting targets are crucial entities for the inhibition of cellular expansion in cancerous cells, leading to several biological and clinical outcomes like cell cycle arrest, apoptosis, and anti-metastatic effect. DNA-interacting targets can be proteins, enzymes, or alkylating/intercalating agents or cellular pathways that occupy binding sites on DNA. The distinctive groups of these targets constitute topoisomerases, DNA Polymerases, Poly ADP-Ribose Polymerase (PARP), and epigenetic regulators. Historically, DNA gyrase (a type II topoisomerase found only in bacteria) was identified as the cellular target of the bacterial DNA gyrase inhibitors [45]. Since then, sustained investigations have established human DNA topoisomerases, hTop1 and hTop2, as important targets for many cancer treatments.

**Figure 4:**
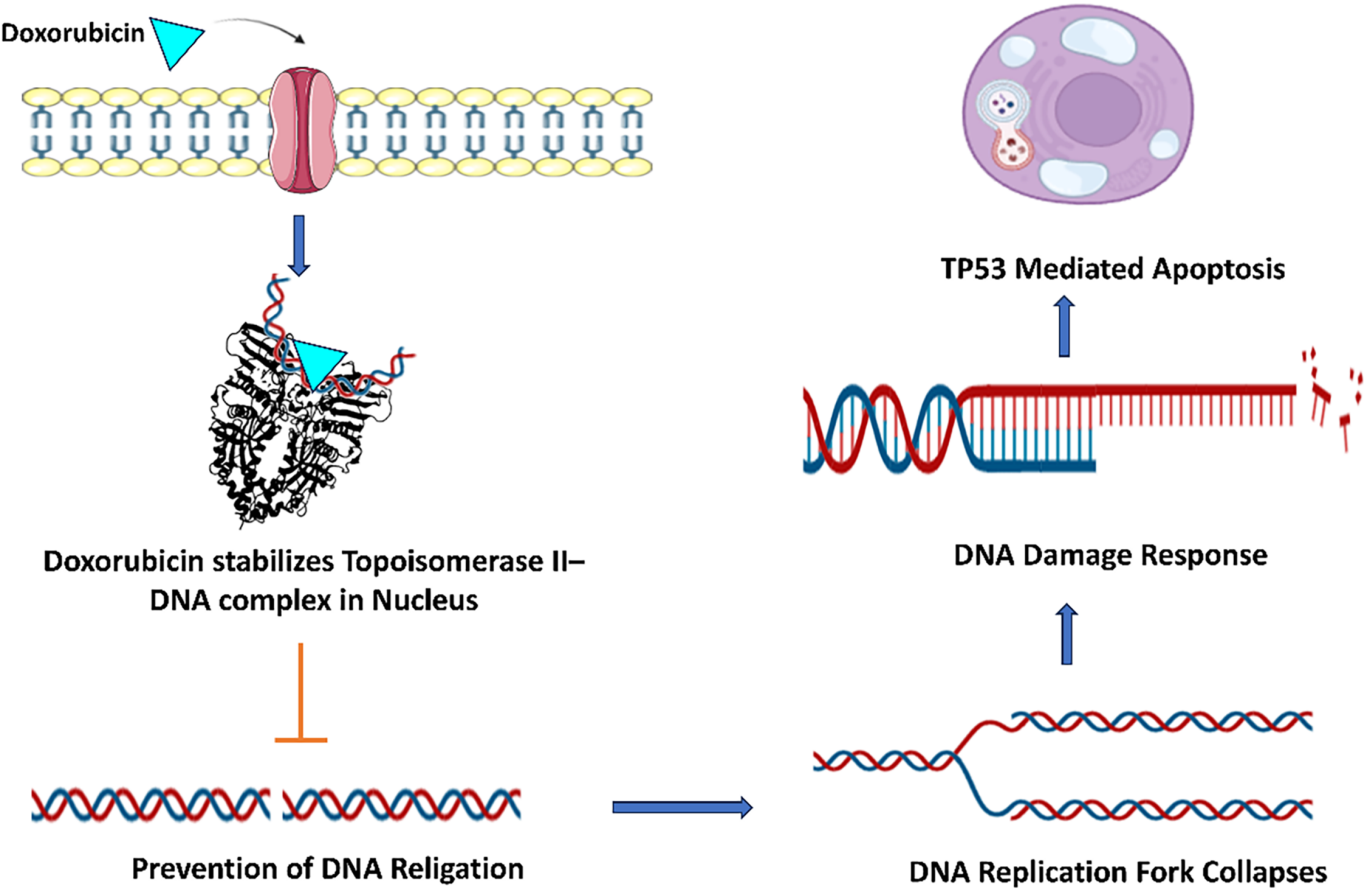
Doxorubicin penetrates the cell membrane and stabilizes the Topoisomerase II-DNA complex within the nucleus through direct binding with DNA. This agent intercalates between the base pairs of DNA, inhibiting Topoisomerase II from resealing DNA breaks. Consequently, it induces double-strand breaks (DSBs) in chromatin, which are subsequently detected by the MRN complex. The signaling cascade initiated by ATM kinase and the phosphorylation of H2AX histone activate the DNA damage response. Ultimately, p53 facilitates the transcription of pro-apoptotic genes, resulting in p53-mediated apoptosis. Created with BioRender.com. Information adapted from the Reference [46,53]

A significant number of small molecules have been identified as inhibitors of hTop1 and hTop2 enzymes. Researchers have successfully manipulated these enzyme activities to achieve therapeutic effects by reprogramming enzymes to exert cytotoxic effects. This therapeutic cytotoxicity is achieved by the formation of a ternary protein-DNA-drug complex that stabilizes the transient DNA break by locking the enzyme–DNA complex. The drug dexrazoxane exerts cytotoxicity using an analogous mechanism of enzyme–DNA complex, leading to DNA strand breaks and ultimately cellular death [46]. The camptothecin-derived agents and anthracyclins like doxorubicin (Fig. 4) are chemotherapeutic drugs following the same mechanism.

Another strategy is inhibiting DNA replication with DNA-damaging agents that modify nucleic acids, rendering them ineffective substrates for DNA polymerase [47]. Chemotherapeutic agents such as temozolomide and cisplatin alter the composition and structure of DNA, thereby downregulating the synthesis and preventing cellular proliferation. The mechanism involves DNA polymerase binding to the DNA template, followed by binding of a deoxynucleoside triphosphate molecule. The polymerase undergoes a structural rearrangement to align the dNTP correctly. The rearrangement enables phosphoryl transfer and another conformational change. In the following step, pyrophosphate is released from the active site after bond formation, leading to polymerase dissociation.

The significant role of PARP, a nuclear enzyme in DNA repair machinery of a cell, makes it an important target for oncological treatments. The underlying mechanisms for DNA repair constitute the non-homologous end joining pathway, the base excision pathway, and nucleotide excision repair. A targeted therapy inhibiting the expression of PARP enzyme by small molecule inhibitors is a promising strategy to suppress cell proliferation, differentiation in tumors. PARP inhibition has a significant oncolytic activity selectively on tumor cells with TMPRSS2-ERG translocation detected in prostate cancers [48]. A significant limitation is that although pharmacologic inhibition demonstrates efficacy in preclinical studies on cell lines and embryo models, its translation to clinical practice remains uncertain. This limitation underscores the necessity for conducting robust clinical trials. Additionally, EWSR1-FLI1 translocations in Ewing’s sarcoma also demonstrate similar cytotoxic effects in malignant tumors [49]. Veliparib, a prominent PARP inhibitor, is potentially exploited as a combination therapy with other anti-cancer drugs like Mitomycin C, FOLFIRI, Paclitaxel, Cisplatin, and Irinotecan hydrochloride.

In recent studies on cases of chronic and acute myeloid leukemia cells [50], selective DNA-PK catalytic inhibitors (e.g., AZD7648, peposertib/M3814) are under investigation. A new class of DNA damage–response (DDR) targets includes Polθ, PARP1/2, DNA-PKcs and PARG, RAD51, WRN, POLQ helicase. Inhibition of these proteins leads to lethality in tumors while sparing normal tissues [51].

Several recent studies have explored possibilities for targeting metabolic pathways in cancer treatment. These studies examine how data is collected, converted into relevant information, and applied to cancer cell metabolism. Researchers have proposed a conceptual framework [52] for integrating metabolic and epigenetic regulatory networks (MER-Net) to guide future research in metabolomics and epigenomics for cancer treatment. However, these high-throughput methods are still in early development and require significant work before they can be applied in practical models.

## 3 Emerging Drug Targets

Over the years, the landscape of novel oncological drug targets has evolved due to Cutting-edge discoveries. Recent advancements in cancer treatment have introduced new targets that address previous limitations, placing a greater emphasis on minimizing side effects for patients. Emerging targets such as Kirsten Rat Sarcoma viral oncogene homolog (KRAS), Claudin 18.2 (CLDN18.2), Trophoblast cell-surface antigen 2 (TROP2), Lymphocyte-activation gene 3 (LAG-3), T-cell immunoglobulin and mucin-domain containing-3 (TIM-3) are outlined as follows.

### 3.1 KRAS G12C

**Figure 5:**
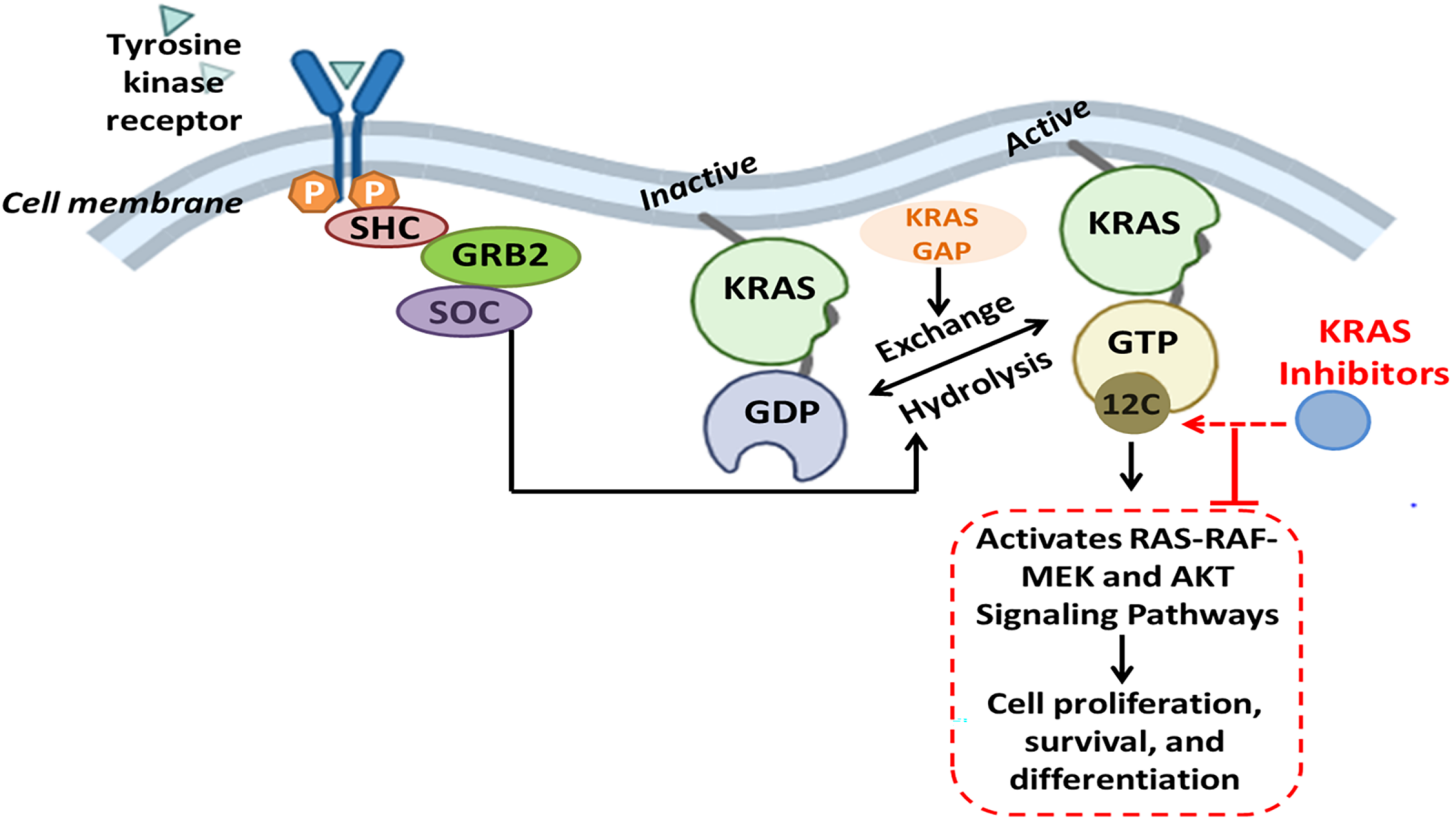
The receptor tyrosine kinases (RTKs) activate adaptor proteins such as SHC, GRB2, and SOS, promoting the exchange of GDP for GTP on KRAS when stimulated by extracellular signals. In its active GTP-bound form, KRAS initiates downstream signaling via the RAS-RAF-MEK and PI3K-AKT pathways, promoting cell proliferation, survival, and differentiation. The KRAS G12C mutation leads to the replacement of glycine with cysteine at codon 12, facilitating covalent interaction with inhibitors that specifically target KRAS G12C. These inhibitors selectively target the mutant KRAS in its inactive GDP-bound state, preventing activation and oncogenic signaling. Created with BioRender.com. Information adapted from the Reference [55]

The KRAS G12C mutation is a recurrent driver alteration in cancer biology, especially lung adenocarcinoma, and is significant for therapeutic targets owing to its biochemical properties [21]. Essentially, KRAS predisposes an oncogenic mutation in lung adenocarcinoma, pancreatic ductal adenocarcinoma, and colorectal cancer. Within these cancer forms, the G12C mutation develops in a specific and significant subtype. This mutation further contributes to tumor onset and progression by maintaining downstream signaling pathways that control cellular proliferation and survival. In lung adenocarcinoma, KRAS G12C is the most frequent molecular subclass, highly associated with smoking, while other cases found in nonsmokers may be explained by passive smoking [54]. Unlike other KRAS mutations such as G12D and G12V, which act as “mini-drivers” demanding additional oncogenic cooperation, G12C acts as a primary driver oncogene. It leads to amplification of lung metastases, but mitigates liver involvement, and treatment sensitivity to bevacizumab and bisphosphonates. Furthermore, tumors bearing this mutation may harbor elevated levels of tumor mutational burden and PD-L1 expression, thereby modulating potential responsiveness to immunotherapies [55]. KRAS G12C, at the molecular level, alters normal GTPase activity by impeding GTP hydrolysis due to suboptimal interactions with GTPase-activating proteins (GAPs). This alteration results in the constitutive activation of KRAS [56]. The persistent activation continuously transmits signals downstream through pathways such as RAS-RAF-MEK and AKT. The G12C mutation changes residue 12 to cysteine, which creates a unique allosteric site for the design of covalent inhibitors, such as sotorasib and adagrasib. The drugs bind irreversibly to the cysteine residue in the switch-II pocket, thereby locking the KRAS protein in the inactive GDP-bound state, inhibiting the signaling from operating downstream, as highlighted in Fig. 5. However, this therapy has certain limitations. The tendency to develop resistance, bypass signaling (EGFR, SHP2), and co-mutations such as KEAP1, STK11, reduces the efficacy of immunotherapy [57]. Results among KRAS mutant lung adenocarcinoma and KRAS wild type lung adenocarcinoma are not in proximity. It necessitates further investigations about KRAS as an oncological drug target. To overcome these resistances, combinations of KRAS with EGFR inhibitors and new ON-state inhibitors such as RMC-7977 [58] are being investigated. RAS inhibitors together with immune-based treatments elicit stronger anti-tumor response in pancreatic cancer models.

### 3.2 Claudin-18.2

Claudin-18.2 (CLDN18.2), a member of the claudin family, plays an important role in the integrity of epithelial tight junctions (TJs) and paracellular permeability to maintain cell polarization and barrier functions in epithelial tissues. The transmembrane protein is encoded by the CLDN18 gene located on chromosome arm 3q22. This gene produces two isoforms, CLDN18.1 and CLDN18.2, through alternative splicing. The latter isoform is predominantly expressed in differentiated epithelial cells of the gastric and pulmonary epithelium. CLDN18.2 consists of four transmembrane domains (TMDs), two extracellular loops, and a cytoplasmic tail, allowing it to interact with intracellular signaling molecules and structural proteins [22].

Misexpression of CLDN18.2 is maintained during the gastric metastatic process, and there is no significant difference in expression between the primary and metastatic lesions [22,59]. In addition to GC, aberrant CLDN18.2 expression is also described in pancreatic, biliary, esophageal, ovarian, and non-small-cell lung cancers. The differences in expression profiles could be the result of differences in immunohistochemistry (IHC) antibodies used in the studies [22]. Functionally, CLDN18.2 dysregulation could affect the integrity of epithelial barriers due to altered chloride flux and ion selectivity, contributing to tumorigenic changes. Further studies generalized that CLDN18.2 mediates downstream signaling through key signaling pathways, including EGFR/ERK, SPAK-p38 MAPK, and protein kinase C (PKC). These findings support that CLDN18.2 plays a multifaceted role in cancer biology [60]. As a prognostic marker, its overexpression has been associated with poor outcomes in some gastric cancer studies, but the results in published literature are nonspecific and inconsistent [59,61].

**Figure 6:**
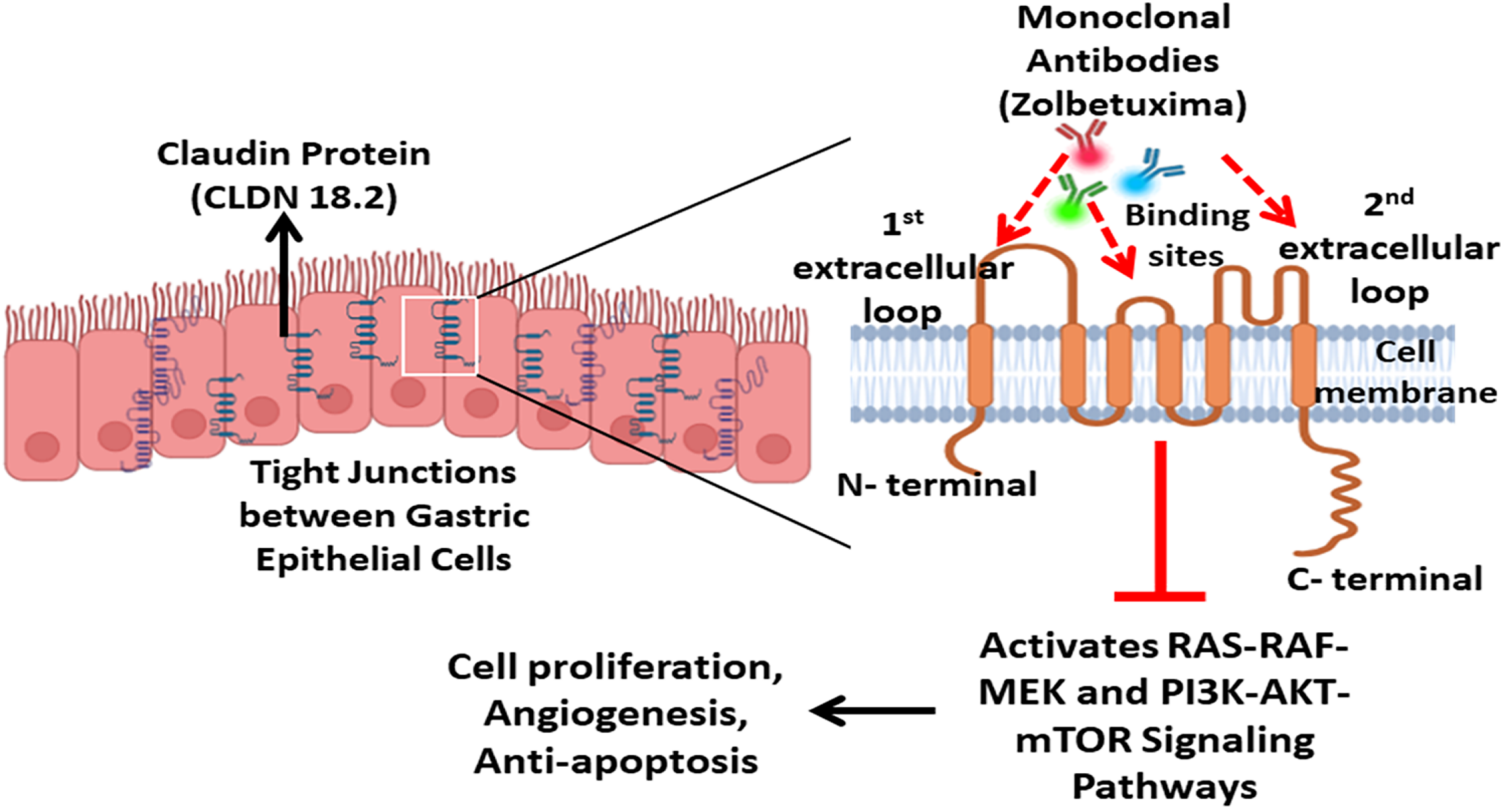
Claudin 18.2 (CLDN18.2), a tight junction protein selectively expressed in gastric epithelial cells, plays a key role in cell signaling pathways that regulate cell proliferation, angiogenesis, and anti-apoptosis. Monoclonal antibodies such as Zolbetuximab bind to the first and second extracellular loops of Claudin 18.2, thereby inhibiting activation of oncogenic RAS-RAF-MEK and PI3K-AKT-mTOR signaling pathways. This blockade disrupts the tumor cell survival and proliferation, representing a targeted therapeutic approach in Claudin 18.2–positive gastric cancers. Created with BioRender.com. Information adapted from the Reference [67]

Zolbetuximab, a monoclonal antibody (MoAb) of the immunoglobulin G1 (IgG1) type, specifically binds to highly expressed CLDN18.2 present on the surface of gastric cancer cells and esophagogastric junction adenocarcinoma (Fig. 6). This drug triggers the immune system to kill these cancer cells through Antibody-Dependent Cellular Cytotoxicity (ADCC) and Complement-Dependent Cytotoxicity (CDC) mechanisms. The phase II trials presented promising results of combination therapy of Zolbetuximab and chemotherapy in patients with CLDN18.2-positive advanced Gastric cancer/Esophagogastric Junction Adenocarcinoma (GC/EGJA) [62].

Bispecific antibody Q-1802 potentially targets both the tumor-specific CLDN18.2 and the immune checkpoint PD-1, inducing both innate and adaptive immunity in the tumor microenvironment [63]. CLDN18.2-directed CAR-T cells are also gaining attention in managing gastric malignancies and have shown promising results in clinical trials [64]. The treatment landscape for patients with gastric cancer is rapidly evolving with combination therapies involving CLDN18.2, chemotherapeutic agents, and anti-PD-1 agents.

Recent advancements in monoclonal antibody Zolbetuximab have shown substantial clinical results and clinical advantage in patients with CLDN18.2-positive gastric and gastroesophageal junction cancers [65]. The debilitating treatment toxicity associated with this regimen, such as nausea, vomiting, neutropenia, and myelosuppression, can be endured with its restricted therapeutic use. Another strategy to overcome resistance constitutes a combination of therapies, including immune checkpoint inhibitors and HER2-directed therapies. Additionally, new modalities, like CD3 bispecific antibodies and CLDN18.2-directed CAR-T cells, are under development [66] to enhance efficacy and overcome resistance. These approaches are continuously undergoing clinical trials, and CLDN18.2 is expected to become a key component of precision oncology.

### 3.3 TROP2

Trophoblast cell-surface antigen 2 (TROP2), encoded by the tumor-associated calcium signal transducer 2 (TACSTD2) gene, is a transmembrane glycoprotein that has gained recognition for its relevance in advanced metastatic cancers. While TROP2 does not fall into the category of an oncogene, its overexpression is often correlated with primary tumor evolution, increased metastasis, and negative prognosis in clinical practice. TROP2 functions in a multifaceted manner in cancer biology. TROP2 has context-dependent impacts on cell proliferation and apoptosis, as it promotes proliferation in some tumors while inhibiting the same in others, and it may have either pro- or anti-apoptotic roles [23,68]. The extranodal nasal NK/T cell lymphoma, gliomas and glioblastomas, and pituitary adenomas exhibit overexpression of TROP2. Disruption of signaling cascades like cyclooxygenase-2 and tumor necrosis factor-α in colon carcinoma cells, PTEN and lipoxygenase in prostate cells, and TGF-β in Langerhans cells can also be attributed to the modulation of TROP2 expression in malignant cells [69].

Several studies have reported an association between TROP2 and mesenchymal phenotype, suggesting that the link between TROP2 and EMT may be cancer type/cell context dependent. TROP2 influences EMT through signaling pathways like PI3K/AKT, NF-κB, and β-catenin signaling [69]. The interactive effects of TROP2 on the signaling pathways are cancer type-specific; for example, TROP2 inactivation will increase AKT signaling in lung adenocarcinoma, but TROP2 in cervical cancer inhibits IGF-1R signaling and ALK inhibition.

TROP2 is clinically relevant as a therapeutic target and is particularly suitable for antibody-based therapies. Combination therapy incorporating chemotherapy, immunotherapy, along with monoclonal antibodies, bispecific antibodies, and antibody-drug conjugates (ADCs) has been the focus of ongoing research. The hRS7, a humanized monoclonal antibody (mAb) of the IgG1 type, has strong receptor interaction for TROP2 in various cancer types, establishing it as a potential broad-spectrum therapeutic antibody [70]. The application of pretargeted radioimmunoimaging utilizing TF12, a bispecific antibody, in conjunction with the radiolabeled di-HSG peptide IMP288 in murine models facilitated the evaluation of this conjugate as a pretargeted antibody–peptide system. TROP2 efficiently binds with TF12, leading to high uptake of di-HSG peptide IMP288 in all subcutaneous PC3 tumors. TF12-mediated uptake in the tumor was substantiated by elevated blood levels in the presence of the radiolabeled compound and improved tumor-to-blood ratios. This study established pretargeted immunoPET with TF12 and 68Ga-labeled IMP288 as a sensitive and rapid imaging method for prostate cancer and its further exploration for drug targets in cancer therapy [71].

**Figure 7:**
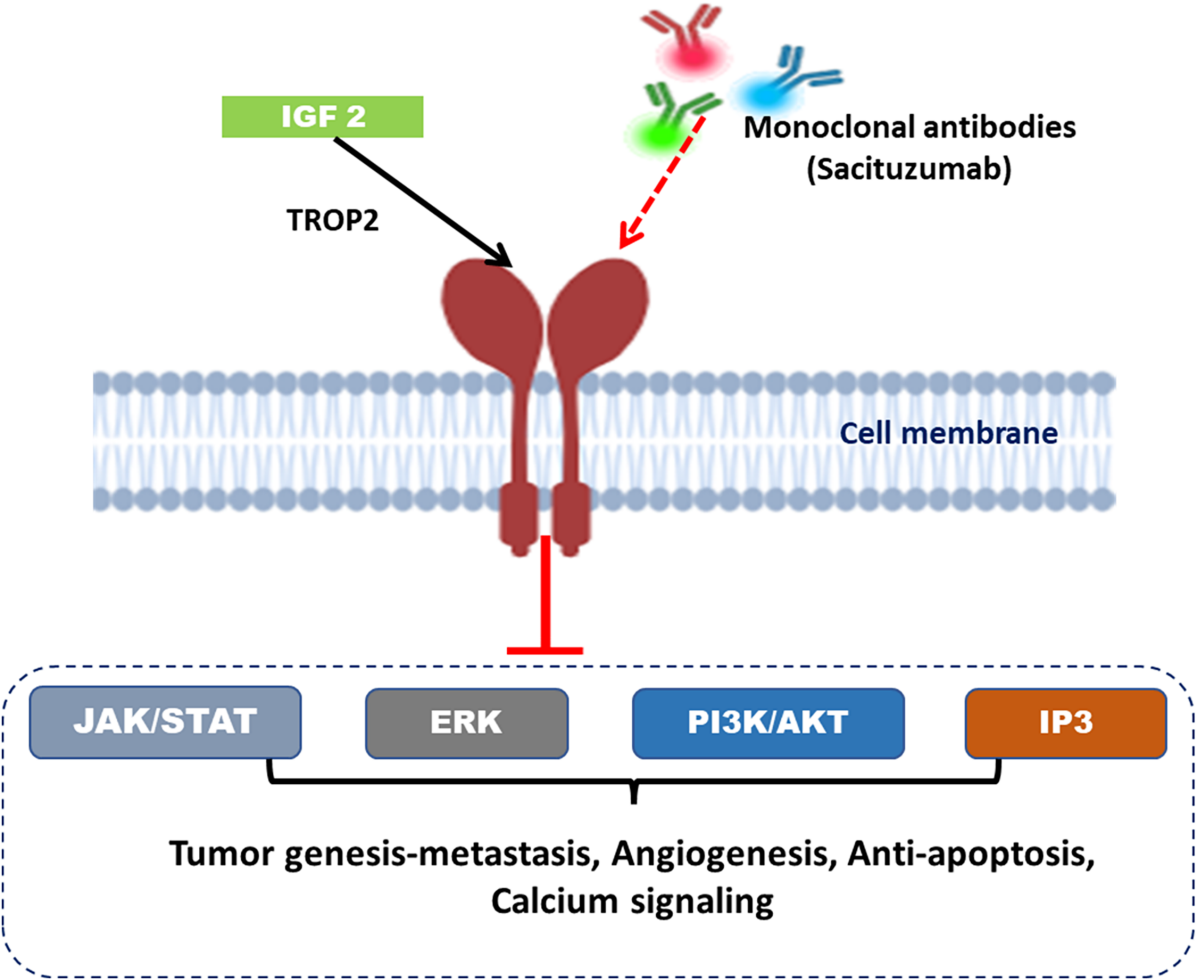
Insulin-like growth factor 2 (IGF-2) binds to TROP2, activating downstream intracellular signaling pathways such as JAK/STAT, ERK, PI3K/AKT, and IP3, which promote tumor progression, metastasis, angiogenesis, anti-apoptosis, and calcium signaling. Monoclonal antibodies like Sacituzumab target Trop-2 on the cell membrane, blocking this signaling and inhibiting associated tumor functions. Created with BioRender.com. Information adapted from the Reference [73]

Some other TROP2-directed antibody-drug conjugates (Fig. 7), like Datopotamab deruxtecan (Dato-DXd), Sacituzumab govitecan (IMMU-132), and RN927C (also known as PF-06664178), significantly suppressed tumor growth in mice and improved their survival rate. However, several underlying limitations [72] include variable TROP2 expression across tumors, development of resistance mechanisms, and off-target toxicities such as neutropenia and diarrhea. Several clinical trials are evaluating Sacituzumab govitecan in other solid tumors, including HR+/HER2-breast cancer and non-small cell lung cancer (NSCLC). Alternatively, the combination of TROP2 with immunotherapy and other targeted agents is also being explored to enhance efficacy and overcome resistance [73].

### 3.4 TIM-3 and LAG-3

The mechanisms by which cancer evades the immune system have garnered significant interest among researchers, with TIM-3 playing a pivotal role in these processes. TIM-3 functions as a co-inhibitory receptor found on various immune cells, including T cells, natural killer (NK) cells, and myeloid cells [24,74]. The expression of TIM-3 is especially obvious in exhausted T cells in the tumor microenvironment (TME), with the TIM-3 receptor conferring lower immune activity and encouraging tumor development [24,75]. The elevated levels of TIM-3, associated with more advanced stages of cancer and poor survival, have been observed in people with ovarian cancer specifically [75]. Mechanistically, it has been suggested that TIM-3 limits the ability of CD4^+^ T cells to become activated through the IL-6-STAT3 signaling pathway, impeding the ability of CD4^+^ T cells to polarize into Th1 and promoting tumor growth [24]. It has also been reported that TIM-3 undermines dendritic cell (DC) capability to become activated by altering nucleic acid sensing and the activation of the inflammasome, which limits immune activity resulting from chemotherapy-induced injury due to the lack of DC function [74].

Importantly, TIM-3 can also be expressed on cancer cells themselves, which means that it may play a role in cancer cell survival and proliferation. The fact that TIM-3 is co-expressed with PD-1 suggests that these different immune factors may have synergistic effects in combination therapies. In fact, preclinical studies showed that TIM-3 blockade could complement the effects of an anti-PD-1 and anti-CTLA-4 strategy even in human cancers [24]. Clinical trials targeting TIM-3 as a single agent or in combination with other therapies are under investigation [24,75].

LAG-3 is similarly a checkpoint receptor expressed mainly on immune cells, namely T and NK cells [24,74]. LAG-3 primarily binds to MHC class II, and its expression on tumor-infiltrating lymphocytes (TILs) is generally associated with poor prognoses [24] although there are some conflicting reports also available in the literature [75]. These therapies also confer some limitations, such as overlap in inhibitory pathways, immune-related side effects, and expression variability across tumors.

**Figure 8:**
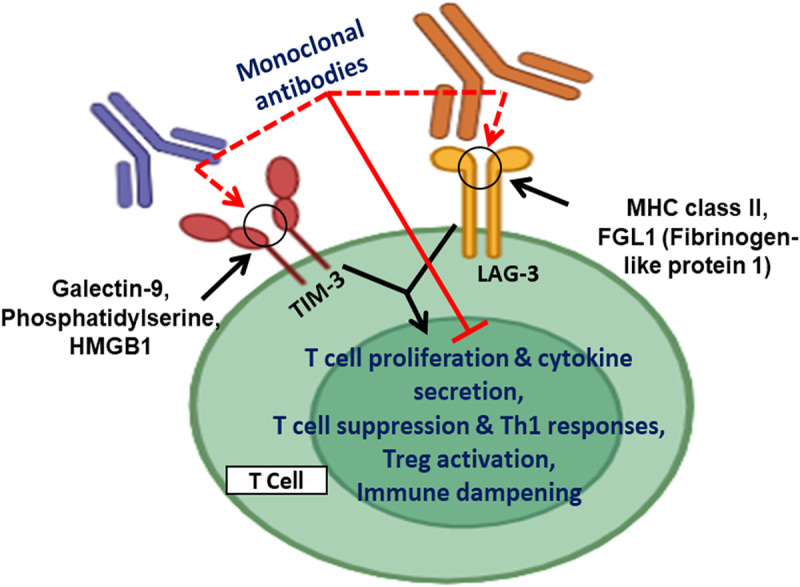
TIM-3 and LAG-3 are inhibitory immune checkpoint receptors expressed on activated T cells. TIM-3 interacts with ligands such as galectin-9, phosphatidylserine, and HMGB1, while LAG-3 binds to MHC class II and fibrinogen-like protein 1 (FGL1). Engagement of these checkpoints suppresses T cell proliferation, cytokine secretion, and Th1 responses, while promoting Treg activation, leading to immune dampening. Monoclonal antibodies targeting TIM-3 and LAG-3 can block these interactions, restore T cell function, and enhance antitumor immunity. Created with BioRender.com. Information adapted from the Reference [78]

Functionally, LAG-3 is known to inhibit T cell proliferation, augment the functioning of regulatory T cells, and inhibit antigen presentation by dendritic cells [24]. Significantly, LAG-3, just like TIM-3, is co-expressed with PD-1, and is a suitable target for combination therapies with immune checkpoint inhibitors, and is currently in clinical trials [24,75]. Fig. 8 enumerates immune checkpoint regulation by TIM-3 and LAG-3 and their therapeutic targeting.

LAG-3 inhibitors such as relatlimab in combination with nivolumab have been reported to improve progression-free survival in melanoma, leading to FDA approval for this therapy [76]. The second wave of immunotherapy targets TIM-3, T cell immunoglobulin, and LAG-3 are demonstrating promising prospects for treating hematologic malignancies and solid tumors [77]. Coformulated Favezelimab/Pembrolizumab (MK-4280A) has completed the clinical trial (NCT05600309) for colorectal cancer and Hodgkin lymphoma (HL) treatment.

Despite the encouraging clinical potential of LAG-3, the physiological function and mechanism of action in tumors are still not well understood. Continued clinical trials are trying to determine how pharmacotherapy can be optimized by adjusting the dosing, combination, and patient selection.

### 3.5 Epigenetic EZH2 and HDACs

EZH2 and HDACs are both epigenetic regulators linked to the development and progression of cancers. However, they differ in mechanisms of action and could be targeted by different therapeutic strategies. EZH2, enhancer of zeste homolog 2, is a central component of the Polycomb Repressive Complex 2 (PRC2), which has relevance in the progression of synovial sarcoma, a highly aggressive soft tissue cancer. A report documents that the fusion oncogene (SS18-SSX) causes either an inactivation of the SWI/SNF complex, inhibition of EZH2, or directly recruits PRC2 to silence important tumor suppressor genes with dysregulated histone modifications [79]. It is indicated that EZH2 is overexpressed in a significant proportion of human synovial sarcoma samples, specifically in 76% of cases, thereby confirming its pathological relevance [80]. Additionally, cancer therapy with a selective EZH2 inhibitor EPZ005687 also exhibits significantly similar antiproliferative and antimigratory effects. Mechanistically, EZH2 mediates its onco-genic effect through histone methylation of histone H3 lysine 27 (H3K27me3), which marks transcriptional repression. The inhibition of EZH2 leads to decreased levels of H3K27me3, re-activating silenced genes that encode growth and migratory pathways. These findings bolster the active clinical development of EZH2-targeted therapies for synovial sarcoma [80].

To access transcriptional events, acetylation is an important step in unwinding closely coiled heterochromatin, which plays a major role in increasing the inner pore space between histones and chromatin. Histone Deacetylases (HDACs) and their antagonistic enzyme families Histone Acetyltransferases (HATs), are crucial in regulating histone acetylation status and consequently the gene expression. HDAC activity, comprising crucial chromosomal protein fusion and carcinogenic events, is manifested in several tumors. Prominent biological functions of HDAC include DNA damage repair, metastasis, autophagy, and transcription [81]. There are 18 human HDACs, which are grouped into four classes: Class I, II, III, and IV, further divided into their subgroups. Class I consist HDAC1, HDAC2, HDAC3, and HDAC8. Class IIa comprises HDAC4, HDAC5, HDAC7, and HDAC9, while Class IIb includes HDAC6 and HDAC10. Class III, also known as the sirtuins (SIRTs), includes SIRT1-7, whereas class IV includes only HDAC11 [82].

Histone deacetylase 2 (HDAC2) has emerged as a critical epigenetic regulator in the survival and proliferation of brain tumor stem cells, particularly in glioblastoma, the most aggressive brain cancer form [83]. HDAC2 acts through complex interactions with SMAD3 and SKI, components of the TGF-β signaling pathway. This epigenetic regulation has significant implications in maintaining the tumorigenic potential of BTSCs in complementary experimental approaches.

A recent study demonstrated that the inhibition of HDAC activity by romidepsin, which involved the modulation of gene expression through either silencing or overexpression, identified HDAC2 as a promising candidate for sustaining the growth and self-renewal characteristics of brain tumor stem cells (BTSC) [84]. The study further emphasized the combined role of HDAC2 and SMAD3 to regulate the balance between self-renewal and differentiation. The involvement of SMAD3 in TGF-β signaling, leading to modulation of the expression of SOX2, GFAP, BDNF, and SKI genes, underscores its importance in epigenetic regulation and tumor behavior. Entinostat and Vorinostat (SAHA) are some iHDAC (benzamide derivatives, hydroxamic acid derivatives, respectively), undergoing clinical trials for brain cancer treatment. Vorinostat has cleared clinical trials, while the same is pending and in the final stage for Entinostat. Clinical evaluations of combination therapies [85] are instrumental in broadening the therapeutic indications for solid tumors, with a particular focus on castration-resistant prostate cancer. This approach aims to enhance the range of treatment options available for patients. Table 2 enumerates some potential drug targets undergoing different clinical trial phases for targeted therapy in cancer management.

**Table 2:** Mechanism of action of some novel drug targets with their delivery routes

Target	Drug class	Drug name	Mechanism of action	Route	Clinical trial phase	Reference
KRAS G12C	Covalent KRAS inhibitor	Sotorasib (AMG510)	Covalent inhibitor	Oral	FDA Approved	[87,88]
		Adagrasib (MRTX849)	CNS-penetrating inhibitor		Phase III	
CLDN18.2	Monoclonal antibody	Zolbetuximab	Monoclonal antibody	IV infusion	Phase III	[89,90]
	Bispecific antibody	AMG 910	Engages T cells via CD3 binding			
TROP2	Antibody-drug conjugate (ADC)	Sacituzumab govitecan	ADC with SN-38	IV infusion	FDA Approved	[91]
TIM-3	Checkpoint inhibitor	Sabatolimab (MBG453)	Blocks TIM-3 to reverse T-cell exhaustion	IV infusion	Phase II/III	[92,93]
	Anti-TIM-3 antibody	Sym023	TIM-3 inhibition in combo with PD-1/LAG-3		Phase I	
LAG-3	Immune checkpoint inhibitor	Relatlimab	LAG-3 blockade combined with nivolumab (PD-1)	IV infusion	FDA Approved	[94]
HDACs	HDAC inhibitor	Vorinostat	Pan-HDAC inhibitor	Oral	FDA Approved	[95]
		Valproic acid	Class I HDAC inhibition	Oral/IV	Phase II	
E2H2	EZH2 inhibitor	Tazemetostat	Inhibits EZH2 methyltransferase activity	Oral	FDA Approved	[96]

These observations highlight HDAC2 as a tractable potential therapeutic target in glioblastoma, as well as the promise of precision therapy with epigenetic interventions.

Transcriptomics and epigenomics are continuously gaining momentum in uncovering the molecular mechanisms of drug resistance and synergy in T-cell lymphoma. Recent findings [86] have indicated that the synergistic effect of EZH2 and HDAC inhibitors is due to increased activity of STAT1 and the downregulation of replication proteins, such as ORC1. The investigation of RNA sequencing (RNA-seq) and Chromatin Immunoprecipitation sequencing (ChIP-seq) facilitated elucidation of the epigenetic mechanisms that underlie drug response and resistance.

## 4 Mechanism of Resistance

Regardless of the advancements of available treatment regimens, doctors still face a common challenge that many cancers eventually become resistant to therapy. In some cases, cancer cells not only resist one treatment but also become unresponsive to several other drugs, a phenomenon known as multidrug resistance (MDR). This adverse outcome is a major setback against routinely administered standard therapies and one of the leading reasons for oncological relapse and fatality [97]. The resistance arises because MDR genes (MDR1 and MDR2) encode P-glycoprotein, which acts as an efflux transporter and pumps out several chemotherapeutic agents, including anthracyclines and vinca alkaloids, thereby reducing their effectiveness in cancer cells. Various studies substantiate that initially, MDR genes are sensitive to cancer treatment. But gradually, the cells develop resistance to chemotherapy by overexpressing MDR genes, releasing a large amount of P-glycoprotein in the intratumoral compartment [98].

**Figure 9:**
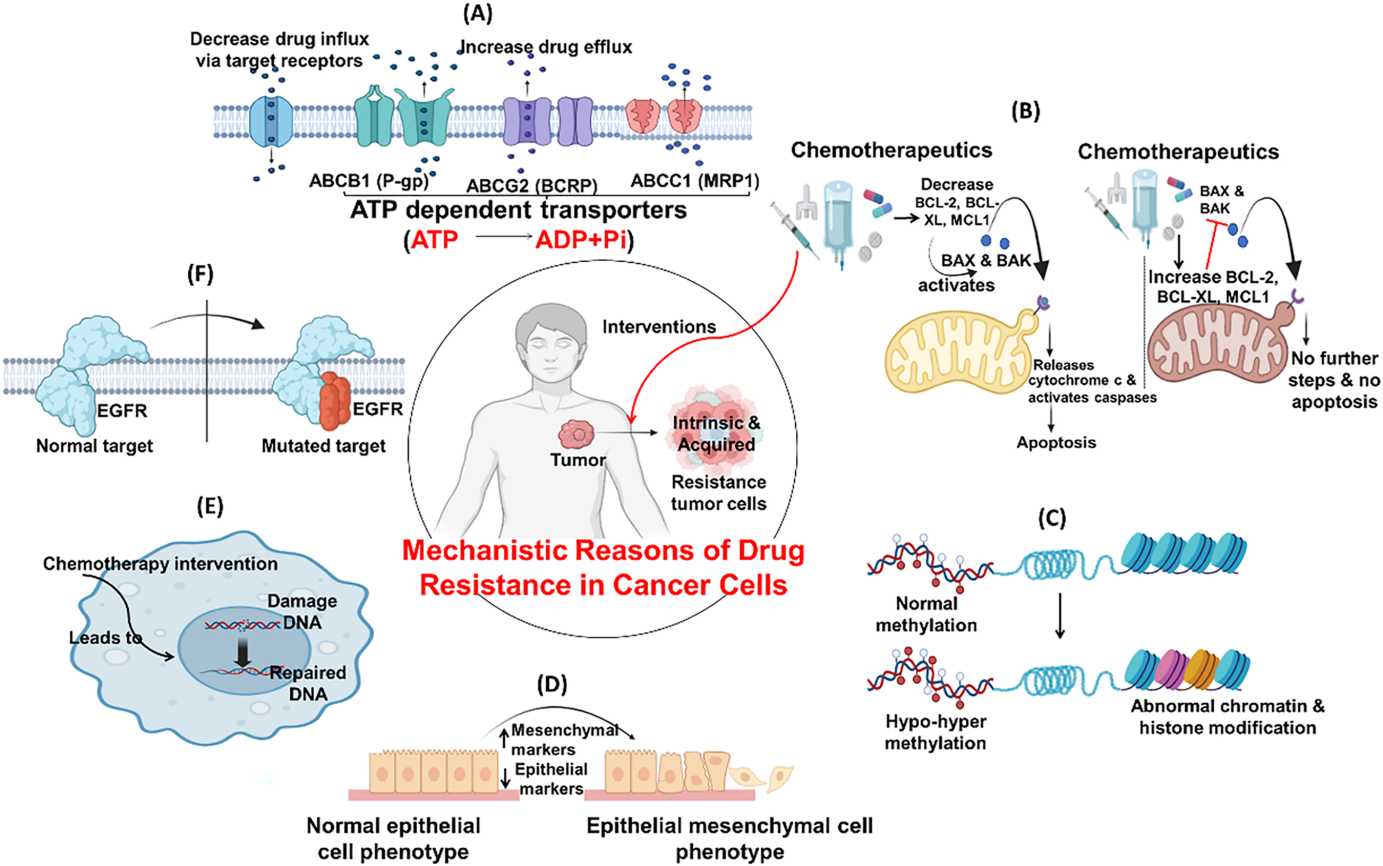
Commonly seen resistance mechanism of chemotherapy. (**A**) Drug influx & efflux via target receptors: Receptors modulates intracellular and extracellular drug concentrations facilitating drug movement, influx through uptake carriers and efflux through P-glycoprotein like pumps; (**B**) Evasion/inactivation of mitochondrial apoptotic pathways: Suppression of pro-apoptotic signals by inhibiting cytochrome c release or upregulating anti-apoptotic factors (e.g., BCL-2, BCL-XL, MCL1), resisting drug-induced cell death; (**C**) Epigenetic changes: Methylation, histone modifications and chromatin remodeling silence tumor suppressor genes or activate drug resistance genes without altering DNA sequence; (**D**) Epithelial Mesenchymal Transition: Loss of epithelial cell markers and gain mesenchymal traits, leads to enhanced motility, invasiveness, and chemoresistance; (**E**) Enhance DNA repair: Upregulation of DNA repair systems rapidly correct drug-induced damage, sustaining cell viability under stress; (**F**) Mutation of cellular targets: Altered molecular structures like EGFR reduce drug binding capacity. Created with BioRender.com. Information adapted from the Reference [102,103]

The cancer therapy resistance is caused by multiple factors, like genetic changes, over-/underexpression of genes, and the environment around the tumor. Some prominent reasons for treatment failure include mutations in drug targets, variation among tumor cells, and cancer cell interactions with the extracellular matrix [99]. These factors enable cancer cells to adapt by either reducing their uptake of the drug, altering their molecular structure, activating alternative survival mechanisms, or evading detection by the immune system [100]. However, advanced treatments, like next-generation drugs, combination therapies, and approaches that target the tumor environment, are showing real promise in cancer management. Clustered Regularly Interspaced Short Palindromic Repeats (CRISPR) and single-cell analysis are becoming popular among health professionals in understanding the disease more precisely, paving the way for more effective, personalized care [101]. Fig. 9 outlines some mechanistic reasons for drug resistance in cancer cells.

Resistance associated with chemotherapeutic, immunotherapeutic, or any other kind of anti-cancer interventions can be classified as intrinsic resistance and acquired resistance. Intrinsic resistance, present from onset, arises from internal tumor factors that help cancer cells survive initial therapy [104]. However, the acquired resistance develops over time as tumors adapt to treatment, reducing the effectiveness of cancer therapies [105]. Table 3 outlines the occurrences of intrinsic and acquired chemoresistance that have developed during treatment with various classes of drugs.

**Table 3:** Drug class, pathway activation, and genetic markers of chemoresistance

S. No.	Drug name	Drug class	Mechanism /Pathway	Cell line & Disease model	Resistance gene/Protein	Chemo resistance type	References
1	Cisplatin, Carboplatin	Platinum-based Alkylating Agent	Enhanced DNA repair	A549 (NSCLC), OVCAR-8, PEO1: PEO4, CP70, CDDP, 2008C13 (Ovarian)	ERCC1, MSH2, BRCA1, BRCA2, NER, HR	Acquired /Intrinsic	[106,107]
2	Gefitinib	EGFR Tyrosine Kinase Inhibitor	Gatekeeper mutation, bypass signaling	NSCLC	EGFR (T790M), MET	Acquired	[108]
3	Trastuzumab	Monoclonal Antibody (HER2)	PI3K/AKT pathway activation	BT/HerR and BT474 (Breast)	PRKAR1A gene and PKA-RIIα PRKAR2A gene	Acquired	[109]
4	Olaparib	PARP Inhibitor	Secondary mutation (HR restoration), DNA repair	High-grade serous ovarian cancers (HGSOC)	BRCA1/2, PALB2, and RAD51	Acquired	[110]
5	Doxorubicin	Anthracycline Chemotherapy	Drug efflux (ABC transporter), Topoisomerase II alteration	HL-60 (Leukemia), MCF-7 (Breast)	MDR1 (ABCB1), TOP2A	Acquired /Intrinsic	[111,112]
6	Paclitaxel, Cabazitaxel, Docetaxel	Taxane Chemotherapy	β-tubulin isotype switching, Drug efflux	MCF-7 (Breast)	TUBB3, MDR1 (ABCB1)	Acquired	[113]
7	Erlotinib, Trametinib	EGFR Tyrosine Kinase Inhibitor	Inhibits EGFR signaling, blocks MEK/ERK pathway	EGFR-mutant metastatic lung adenocarcinoma	EGFR T790M, BRAF	Acquired	[114]
8	Vemurafenib	BRAF Inhibitor	Alternative splicing, MAPK pathway activation	A375 (Melanoma)	BRAF (V600E splice), NRAS	Acquired	[115]
9	Imatinib, Dasatinib, Nilotinib, Bosutinib, Ponatinib,	BCR-ABL Tyrosine Kinase Inhibitor	Kinase domain mutation, bypass signaling	Chronic myeloid leukemia (CML)	BCR-ABL	Acquired	[116]
10	Sorafenib, Imatinib, Nilotinib, Dasatinib, Gefitinib, EKI-785, Canertinib, Danusertib	Tyrosine kinase inhibitor	Kinase domain mutation, Drug efflux	Different cancer models	ABCG2/BCRP, ABCB1/P-gp/MDR1, ABCC1/MRP1	Acquired	[117]
11	Etoposide	Topoisomerase II inhibitor	Topoisomerase II alteration, Drug efflux	HL-60 (Leukemia)	Src tyrosine kinase family	Acquired	[118]
12	5-Fluorouracil	Antimetabolite (Pyrimidine Analog)	Target overexpression, DNA repair loss	HCT116 (Colon)	TYMS, HSP90/Src	Acquired	[119]
13	Crizotinib	ALK/ROS1 Tyrosine Kinase Inhibitor	Kinase domain mutation (target alteration)	Tumor tissues from two NSCLC patients and HCC78CR1-3 cells	ROS1 G2032R and L2155S	Acquired	[120]
14	Brigatinib, Lorlatinib, Crizotinib, Ceritinib, Alectinib	ALK Tyrosine Kinase Inhibitor	ALK/EML4/PI3K (Kinase domain mutation, bypass)	EML4–ALK + NSCLC (Variants 1 & 3)	ALK rearrangement—(L1196M, G1269A, C1156Y/C1156T, I1171T/I1171N/I1171S, S1206C/S1206Y, E1210K, L1152P/L1152R, V1180L, I1151T, F1174C/F1174L, F1245C, G1202R, D1203N, L1198F, L1256F, G1202R/S1206Y)	Acquired	[121]
15	Osimertinib	Third-generation EGFR Tyrosine Kinase Inhibitor	EGFR/PI3K/AKT (Gatekeeper mutation, bypass)	NSCLC (exon 19 del, L858R, T790M)	EGFR T790M, C797S	Acquired	[122]
16	Sotorasib, Adagrasib	KRAS G12C Inhibitor	KRAS/MEK/ERK (Target mutation, MAPK reactivation)	NSCLC	KRAS G12C secondary mutations	Acquired	[21]
17	Nivolumab, Pembrolizumab	Anti-PD-1 Immune Checkpoint Inhibitor	Loss of antigen presentation, IFN-γ pathway alteration	Melanoma models	IPRES signature (AXL, TWIST2, WNT5a, MHC I, β2M)	Acquired	[123]
18	Tamoxifen	Selective Estrogen Receptor Modulator	Ligand-binding domain mutation, metabolic inactivation	2 Patients with MBC (Breast)	ESR1 (Tyr537Ser, Glu542Asp, Leu536Arg, Arg548Cys)	Acquired	[124]
19	Ibrutinib	BTK inhibitor	Blocks BCR signaling, chemokine /adhesion pathways	CLL, MCL, DLBCL, WM, MZL	BTK (C481S), PLCG2, CARD11, BIRC3, TRAF2/3, CCND1, XPO1, MYD88	Acquired /Intrinsic	[125]
20	Idelalisib	PI3Kδ inhibitor	Inhibits PI3K/Akt/mTOR pathway	CLL, FL, NHL	MAPK (BRAF/NRAS /KRAS), IGF1R, IL6/STAT5	Acquired /Intrinsic	[125]
21	Acalabrutinib	BTK Inhibitor	Target mutation, Bypass signaling	Chronic lymphocytic leukemia (CLL)	BTK (C481S), PLCG2	Acquired	[126]
22	Venetoclax	BCL-2 Inhibitor	Glutathione metabolism (Drug efflux, apoptosis)	HL-60/ADR, AML models	ABCC1, BCL-2 family	Acquired	[127]

Intrinsic resistance refers to built-in defense mechanisms that tumor cells possess before therapy even begins. Also known as primary resistance, this phenomenon causes an immediate lack of response to treatment, significantly contributing to therapy failure [128]. This resistance is often driven by genetic mutations already present in tumor cells, making certain drugs ineffective from the beginning. Key contributors include genetic alterations that reduce drug sensitivity, activation of drug efflux pathways, and tumor-driving genes that support survival. Tumor-initiating cells also significantly contribute to resisting treatment [129]. On a molecular level, this resistance often involves alterations in drug targets. Unlike intrinsic resistance, acquired resistance arises after an initially successful response to treatment. This form of resistance is particularly challenging because it affects patients who initially show improvement, only to later relapse [128]. Several mechanisms contribute to acquired resistance, including secondary mutations in drug targets such as T790M, C797S mutations in epidermal growth factor receptor (EGFR)-targeted lung cancers, activation of alternative signaling pathways, increased drug efflux transporters, and epigenetic changes that alter gene expression [130]. Resistance can emerge rapidly, with some markers like MDR1/P-glycoprotein increasing within hours, or more slowly through persister cells that survive and develop resistance over time [131].

### 4.1 Mechanism of Resistance in Targeted Therapy

#### 4.1.1 Tumor Heterogeneity in the Case of Intrinsic and Acquired Resistance

Tumor heterogeneity refers to the genetic and molecular diversity among cancer cells within the same tumor. This complexity allows resistant clones to survive therapy and repopulate, making treatment more difficult and encouraging resistance by reshaping the tumor’s local environment [132]. One of the primary mechanisms is clonal evolution, where cancer cells undergo genetic changes over time and adapt responses to the selective stress that is imposed by therapies. This leads to the survival and expansion of resistant subclones, rendering the tumor intractable as it progresses [133]. Furthermore, single-cell sequencing studies have highlighted a high degree of heterogeneity within tumors, suggesting that individual cancer cells can differ greatly in gene expression profiles and structural alterations such as copy number variations. This variability has been observed across multiple cancer types, including those of the lung, liver, brain, and breast [132]. Spatial and temporal heterogeneity is another challenge, and exhibits unique resistance mechanisms [134].

Instead of existing as a uniform mass, tumors are made up of a diverse mix of genetically distinct cells, which creates multiple pathways for resistance mechanisms. This diversity appears in different tumor sites within the same patient (spatial heterogeneity) and can also evolve (temporal heterogeneity) [135]. This complexity helps to explain why some cancer cells are eliminated by therapy while others persist. Previous studies in esophageal cancer patients have shown a fivefold increase in MDR1 gene expression after chemotherapy, reflecting the survival and growth of resistant cells [136]. The interaction between these forms of resistance is believed to contribute to 80%–90% of cancer-related deaths. Addressing both types of resistance is therefore essential for improving long-term outcomes in cancer care.

#### 4.1.2 Mutation in Targets

The alterations induced by mutations may influence the efficacy of drug binding to its intended target, thereby diminishing the effectiveness of the treatment. Mutations in genes like EGFR or ALK can cause patients to stop responding to tyrosine kinase inhibitors (TKIs) in the case of lung cancer. Sometimes these mutations are innate, while at other intervals, these develop over time as the treatment progresses, ultimately leading to treatment failure [137]. Similarly, there is another example of mutation where mutated, overexpressed, or fused tropomyosin receptor kinases (TRKA, TRKB, TRKC) play a role in cancer therapy evasion. TRKs are encoded by NTRK1/2/3 and activated by NGF, BDNF, and NT-3. TRK inhibitors are effective cancer therapies but face resistance, mainly due to mutations in the kinase domain (e.g., G595R, G623R, G667C). Resistance can also arise from MAPK pathway activation, which can be potentially mitigated by combining TRK and MEK inhibitors [138]. Resistant tumors often find pathways for proliferation by target protein modification or activating alternative signaling pathways that help them survive.

(i) Molecular Alterations in EGFR: T790M and C797S Mutations in NSCLC and Their Impact on Drug Targeting via Steric Hindrance

One of the reasons that cancer cells are constantly adapting and that they resist treatment is that they develop genetic changes in the proteins that drugs are designed to target. However, this phenomenon also arises as a result of continuous therapeutic intervention. EGFR is a key receptor in the HER family and plays an important part in regulating cell growth, survival, and differentiation. However, resistance to EGFR inhibitors often develops, which reduces their persistent therapeutic effect. This resistance is typically categorized into on-target resistance and off-target resistance. The on-target resistance occurs due to mutations within the EGFR gene itself. The most common mutation is the T790M mutation, which alters the ATP-binding site of the receptor. This structural change increases EGFR’s affinity for ATP while decreasing its binding to EGFR inhibitors, rendering first- and second-generation tyrosine kinase inhibitors (TKIs) ineffective [25,30]. In case of off-target resistance, the cancer cells bypass EGFR signaling by activating alternative pathways, such as RAS/MAPK or PI3K/AKT, allowing tumor growth to continue despite EGFR inhibition [138]. In case of T790M mutation in the EGFR gene, the single change swaps out one amino acid for another, creating steric hindrance that prevents drugs like gefitinib and erlotinib from binding effectively [139].

Similarly, in anaplastic lymphoma kinase (ALK)-positive non-small cell lung cancer (NSCLC), mutations like L1196M or G1269A reshape the protein’s active site, reducing the efficacy of the second line of ALK inhibitors [140]. Researchers have suggested that resistant tumors often activate downstream pathways like RAS/MEK/ERK, PI3K/AKT, and JAK3-STAT3, bypassing ALK signaling. Resistance also arises due to poor blood-brain barrier penetration of first-generation inhibitors and the emergence of mutations such as G1269A, F1174X, L1196M, and G1202R (common in second-generation resistance). Additionally, MET amplification has also been linked to resistance in some cases [138].

Third-generation inhibitors of EGFR, like osimertinib, were designed to overcome T790M-related resistance. However, new mutations like C797S have been detected in about 10%–26% of patients rendering osimertinib ineffective by disrupting its covalent bond with the EGFR protein [141]. The C797S mutation, located in exon 20 of the EGFR gene, acts as the main resistance mechanism to third-generation EGFR inhibitors, which target the T790M mutation. This missense mutation (cysteine to serine) disrupts drug binding, allowing EGFR to continue signaling by binding ATP [142]. Oxnard et al. [143] revealed that in patients exhibiting drug resistance, the loss of the T790M mutation was associated with a diminished duration of response. This finding suggests that the mutation may play a significant role in contributing to osimertinib resistance [143]. The arrangement of these mutations, or allelic configuration, also plays a significant role in managing resistance for better therapeutic efficacy. If C797S and T790M exist on opposite gene copies (trans), combined therapy may still be effective. But if both are on the same copy (cis), resistance to all current EGFR-targeted treatments typically occurs, limiting therapeutic options.

(ii) Oncogenic Amplification and Bypass Signaling of MET and Resistance to EGFR-TKIs in Cancer Therapy

The Mesenchymal epithelial transition factor (MET) gene, located on chromosome 7, encodes the c-MET receptor, which binds hepatocyte growth factor (HGF) to activate pathways that regulate cell growth [144]. In cancers, MET amplification or mutations can abnormally activate signaling pathways like PI3K-AKT, RAS-MAPK, and Wnt/β-catenin. MET amplification is a known cause of resistance to EGFR-TKIs, as it reactivates downstream signals like ERBB3 and PI3K-AKT, bypassing EGFR inhibition [145]. Specific mutations such as Y1230, D1228, and D1228N also cause resistance to MET inhibitors by altering drug binding or activating MET signaling. Additionally, exon 14 skipping mutations impair MET degradation, leading to persistent activation and drug resistance. These mutations often coexist with EGFR T790M, and combining EGFR and MET inhibitors shows limited success, emphasizing the need for more effective strategies [138].

When targeted drugs target and neutralize the main growth-promoting signal in cancer cells, often alternate pathways are activated. In lung cancer, MET gene amplification occurs in about 20% of NSCLC patients resistant to EGFR inhibitors. It activates ERBB3-related signaling, bypassing the blocked EGFR route, and sustaining tumor growth [139]. The resistance also arises through independent pathways like MAPK, STAT, and PI3K/AKT, allowing cancer cells to survive despite EGFR inhibition [146]. The FLAURA study found MET amplification as the most frequent resistance mechanism in about 15% of patients treated with osimertinib as a first-line therapy [147]. This resistance often coexists with other changes, such as CDK6 or BRAF amplifications, possibly due to a single alteration on chromosome 7q [146].

In chronic myeloid leukemia (CML), BCR-ABL1 mutations like T315I disrupt imatinib binding but retain kinase activity, leading to drug resistance [148]. In melanoma, 19%–28% of melanomas that no longer respond to RAF inhibitors, there is an upsurge in the number of copies of the BRAF V600E mutation. This boosts ERK signaling directly, bypassing CRAF and helping the tumor resist treatment [149].

#### 4.1.3 Tumor Microenvironment

The tumor microenvironment (TME) is generally recognized as a life-threatening determinant of cancer progression and therapeutic resistance. It is not merely a passive background for tumor growth but an active, dynamic ecosystem composed of non-malignant cells such as fibroblasts, immune cells, and endothelial cells, along with a dense extracellular matrix (ECM) and a variety of soluble signaling molecules. Collectively, these components shape the behavior of tumor cells, often facilitating their survival in response to pharmacological interventions [150,151].

(i) Protective Niches and Cellular Dormancy

The TME contributes to drug resistance by forming protective niches that shield cancer cells from chemotherapy through cell–cell and cell–matrix interactions. Additionally, it also induces dormancy, where quiescent tumor cells evade drugs, targeting actively dividing cells, which is a key factor in relapse and minimal residual disease [152]. In bone, mesenchymal stem cells (MSCs) secrete TGF-β2 and BMP7 to maintain disseminated tumor cells (DTC) dormancy and resistance to chemotherapy. In the brain, astrocyte-derived laminin-211 enforces dormancy via YAP inhibition, protecting DTCs from elimination [153].

(ii) Cytokine Signaling, Immune Modulation, and Hypoxia

Fibroblasts and immune cells in TME release cytokines like ILs and TGF-β that activate survival pathways [154]. The TME suppresses immune responses by upregulating PD-L1 and CTLA-4, promoting immune evasion. Tumor-derived extracellular vesicles carrying PD-L1 can suppress T-cell activation in draining lymph nodes, leading to resistance to immune checkpoint blockade [155]. Myeloid-derived suppressor cells (MDSCs) and tumor-associated macrophages (TAMs) express PD-L1 and other inhibitory molecules. In EGFR-mutant NSCLC, osimertinib increases PD-L1 via STAT3, recruiting immunosuppressive cells like Tregs and MDSCs [156]. Myeloid cells (TAMs, neutrophils, MDSCs) secrete immunosuppressive cytokines (IL-10, TGF-β) and metabolites (earginase, IDO) that inhibit T-cell function and promote resistance to immunotherapy. In pancreatic cancer, TAMs promote T-cell exclusion and dysfunction via CXCL12 and TGF-β secretion [57]. Neutrophils with high FATP2 expression produce prostaglandin E_2_, enhancing immunosuppression and resistance to ICB [153,157]. Hypoxia within the TME activates HIF-1α, increasing adenosine to suppress cytotoxic T cells [158]. This biochemical barrier pairs the physical and molecular mechanisms, jointly creating a field or space for tumor cells within the hostile host environment. Dysfunctional, leaky blood vessels impair drug delivery and create hypoxic niches that promote aggressive, therapy-resistant phenotypes. Studies exhibited that anti-angiogenic therapies normalize vasculature and improve drug delivery, but resistance arises via alternative vascularization (vessel co-option, vascular mimicry). Hypoxia-induced expression of PD-L1 on endothelial cells and immune cells further impairs immunotherapy response [153].

(iii) Physical and Biochemical Drug Sequestration

CAF-generated ECM (collagen, hyaluronan) creates physical barriers that trap drugs [153,159] exploited in pancreatic cancer, where gemcitabine diffusion is hindered. CAFs remodel the extracellular matrix (ECM), creating a physical and biochemical barrier that limits drug penetration (cisplatin and doxorubicin) and T-cell infiltration. Moreover, CAFs secrete exosomes carrying miRNAs like miR-21, which can be internalized by tumor cells to enhance their survival and drug resistance [160]. In pancreatic ductal adenocarcinoma, CAF-derived CXCL12 excludes T cells and reduces the efficacy of anti-PD-L1 therapy [161]. NOX-A12, a pegylated L-oligoribonucleotide that binds and neutralizes CXCL12, has shown some early promise in the treatment. However, CXCL12-directed therapies are yet to be investigated in this domain. Tipifarnib is another contender showing potent inhibition of CXCL12 gene expression in human PSCs, as indicated through *in vivo* studies [161]. CAFs in breast and lung cancers secrete IL-6 and IL-8, supporting cancer stem cells and conferring chemoresistance. Even depletion of certain CAF subsets (e.g., myoCAFs) can paradoxically accelerate tumor growth by enriching immunosuppressive iCAFs. To overcome this resistance, PEGPH20 exhibited promising early-phase tolerability and preclinical benefit in phase III clinical trials, yet it has failed to improve progression-free survival and overall survival in Hyaluronan-high metastatic Pancreatic Ductal Adenocarcinoma [162].

#### 4.1.4 Apoptosis Evasion in the Tumor Microenvironment

Resistance is further reinforced by mechanisms that prevent apoptosis, the programmed cell death process critical for eliminating damaged or dangerous cells. Phosphoinositide 3-kinases (PI3Ks) are intracellular lipid kinases made up of a p110 catalytic subunit and a p85 regulatory subunit. They phosphorylate inositol at the 3-position and play a key role in many cancers when dysregulated. Li et al. [163] discovered that in glioma sphere-forming cells resistant to PI3K inhibitors, Aurora-A levels were increased while pCDK1 levels were decreased. Further analysis revealed that the interaction between the Tie2 and FGFR1 receptors in these cells led to overactivation of the Aurora-A/PLK1/CDK1 signaling pathway, which is a major contributor to resistance against PI3K inhibitors [163].

(i) BCL-2 Family Proteins

The BCL-2 family proteins regulate intrinsic apoptosis. Overexpression of anti-apoptotic proteins, such as BCL-2, BCL-XL, and MCL1, blocks mitochondrial membrane permeabilization, preventing cytochrome-C release and caspase activation [164]. This mechanism is prevalent in many types of cancers. In triple-negative breast cancer, up to 80% of MYC-amplified tumors also co-amplify MCL1. Post-treatment samples often show elevated BCL-2, particularly in chemo-resistant HER2-positive tumors [165].

(ii) Caspase-8 Inactivation and Tumor Progression

Aside from the mitochondrial pathway, resistance also develops via defects in extrinsic apoptosis. Caspase-8, essential for the extrinsic apoptotic signaling pathway, is often mutated or downregulated in gastric cancer [166]. These mutations can block TRAIL-induced apoptosis and promote cancer cell migration and invasion, linking resistance to increased tumor aggressiveness [167].

### 4.2 Mechanism of Resistance in Chemotherapy

The cancerous cell employs several intracellular mechanisms to evade the cytotoxicity of chemotherapeutic drugs. These mechanisms include drug efflux and transporter-mediated resistance and molecular adaptations.

#### 4.2.1 Drug Efflux and Transporter-Mediated Resistance

Occasionally, cancer cells survive chemotherapy by actively pumping out the drugs from cellular compartments, a process known as transporter-mediated resistance. The mechanism incorporates ATP-binding cassette (ABC) transporters, a family of proteins that use energy from ATP to remove toxic substances, including chemotherapeutic drugs, across cellular membranes. The action leads to reducing the drug concentration inside the cancer cell, often pushing it to suboptimal levels, thereby allowing the tumor to persist or even thrive.

ABCB1 (P-glycoprotein or P-gp) is an established ABC transporter involved in multidrug resistance (MDR). It possesses more than one domain for drug binding and ATP hydrolysis [168] and can actively eliminate various drugs like doxorubicin, paclitaxel, vincristine, and colchicine from cancer cells [169]. Overexpression of P-gp is responsible for resistance in cancerous cells such as lung, breast, liver, and osteosarcoma, especially under hypoxic conditions [168]. The ABCB1 gene, located at chromosome 7q21, is frequently amplified or shows increased copy numbers in drug-resistant cancer cells and tumor tissues. This genetic alteration results in higher production of the P-glycoprotein (P-gp), thereby boosting its ability to expel chemotherapeutic drugs from cells [170]. Beyond these genomic changes, ABCB1 expression is also tightly regulated at the transcriptional and epigenetic levels. Various signaling pathways, including PI3K/AKT and MAPK/ERK, and transcription factors such as estrogen receptor alpha (ERα) and Nkx-2.5, play crucial roles in modulating its activity [171]. Furthermore, epigenetic changes, structural rearrangements of the gene, and elevated tumor mutational burden also contribute to its dysregulated expression in cancer.

ABCG2 (BCRP) is another key half-transporter with one ATP-binding site and six transmembrane helices [172]. It exports drugs like topotecan and mitoxantrone across cellular membranes [173]. Recent research involving HeLa cells has shown that overexpression of ABCG2 leads to a dramatic increase in drug resistance, up to 15-fold for mitoxantrone and over 5-fold for topotecan. In mouse models, reduced expression of ABCG2 demonstrated improved drug response and survival [174].

ABCC1 (MRP1) exports chemotherapy drugs conjugated to glutathione (GSH) and other detoxified products across membranes [175]. It co-transports drugs such as vincristine and etoposide with GSH [176]. Elevated ABCC1 levels reduce cancer cell sensitivity to BCL-2 inhibitors by limiting intracellular drug retention that was highlighted in ABCC1-deficient mice, where cells presented lower glutathione-related compound levels after stimulation [175].

However, ABC transporters depend on ATP hydrolysis to export drugs, requiring energy from up to two ATP molecules per transport event. Thus, mitochondrial ATP production significantly influences transporter efficiency. In chemoresistant NCI/ADR-RES cells, inhibiting ATP synthesis from the mitochondrial pathway with oligomycin led to increased doxorubicin accumulation, indicating reduced drug efflux [177]. In HEK 293T cells that overexpress ABCG2, the inhibition of mitochondrial ATP production resulted in the retention of Hoechst dye, whereas the inhibition of glycolysis exhibited minimal impact. This observation underscores the critical role of mitochondrial energy in the development of drug resistance.

#### 4.2.2 Molecular Adaptations Promoting Resistance

In addition to the protective roles played by the tumor microenvironment, cancer cells also undergo intrinsic molecular adaptations that protect them against therapeutic assault.

(i) Enhanced DNA Repair Mechanisms

Tumors with BRCA1/2 mutations are initially susceptible to PARP inhibitors or platinum chemotherapy. However, secondary “reversion mutations” can restore homologous recombination (HR) repair by correcting the gene’s reading frame. These occur in up to 30.7% of treated cancers and are linked to resistance across ovarian, breast, prostate, and other cancers, often leading to aggressive relapse and cross-resistance to PARP and platinum agents [178,179]. Patients often develop multiple distinct reversions within the same tumor, and these mutations have been documented in the ovaries, breast, prostate, and other cancers. Clinically, the presence of BRCA reversions is linked with cross-resistance to both PARP inhibitors and platinum agents, often leading to aggressive disease relapse [128].

(ii) Nucleotide Excision Repair and Oxaliplatin Resistance

One more resistance mechanism involves the upregulation of ERCC1, a key component of nucleotide excision repair (NER). In oxaliplatin-resistant tumors, ERCC1 expression is 1.5 times higher than in sensitive ones. This impairs response not only to oxaliplatin but also affects 5-fluorouracil (5-FU) sensitivity due to related repair mechanisms [180].

### 4.3 Alternative Mechanism of Drug Resistance

In addition to well-characterized resistance pathways, emerging evidence highlights several alternative mechanisms that enable tumor cells to evade the effects of anticancer therapies. One such mechanism is vessel co-option observed by Kuczynski et al. [181] a process in which tumor cells hijack existing blood vessels from surrounding tissues rather than relying on angiogenesis. This phenomenon not only undermines the efficacy of anti-angiogenic treatments but also enhances tumor invasiveness. Importantly, vessel co-option is a reversible process, which may further complicate therapeutic targeting [181].

Another key contributor to resistance is chemotherapy-induced genetic evolution reported by Cipponi et al. [182]. Accumulated DNA damage during treatment increases the likelihood of mutations, allowing for the selection and survival of drug-resistant clones. The mTOR signaling pathway plays a critical role in this adaptive process. While mTOR inhibition can suppress tumor proliferation, it may paradoxically promote resistance by facilitating the survival and clonal expansion of tumor cells under selective pressure [182].

Furthermore, another study highlighted that macropinocytosis, a non-selective form of endocytosis, allows cancer cells to engulf extracellular proteins, nutrients, and cellular debris. This pathway becomes especially important in nutrient-deprived tumor environments. In breast cancer models, tumor cells have been shown to utilize macropinocytosis to recycle biomolecules from apoptotic cells, supporting survival under therapeutic stress. Genetic studies have demonstrated that inhibition of macropinocytosis-related regulators, such as CARMIL1-AA, can sensitize cancer cells to chemotherapy agents like 5-fluorouracil [183].

Metabolic reprogramming also plays a significant role in drug resistance. Specifically, dysregulated glycolysis has been implicated in chemoresistance across various cancer types [184]. Overexpression of PTBP1 in colon cancer enhances glycolytic activity and contributes to resistance against drugs such as vincristine and oxaliplatin. Silencing PTBP1 has been shown to restore chemosensitivity by impairing glycolysis [185]. In breast cancer, the adipokine (leptin) promotes endocrine resistance by upregulating estrogen receptor expression, contributing to reduced sensitivity to tamoxifen [186].

Alterations in lipid metabolism are also associated with resistance. Treatment with bevacizumab, for example, has been shown to induce reprogramming of fatty acid oxidation in colon cancer cells, enhancing their capacity to utilize free fatty acids for growth and survival [187]. Additionally, loss of mitochondrial DNA (mtDNA) promotes glycolysis and supports cancer cell viability under stress conditions. This metabolic shift, often accompanied by activation of the AKT/mTOR signaling pathway, further contributes to chemoresistance, particularly in colorectal cancer treated with 5-FU and oxaliplatin [188].

Exosome-mediated communication between resistant and sensitive cells has emerged as a novel mechanism of resistance. In oxaliplatin-resistant colorectal cancer, resistant cells have been shown to transfer ciRS-122, a circular RNA, via exosomes to sensitive cells. ciRS-122 acts as a miR-122 sponge, leading to upregulation of PKM2, a key enzyme in glycolysis. This promotes metabolic reprogramming and enhances drug resistance in recipient cells [189].

## 5 Strategies to Overcome Resistance

Many cancers become resistant because they rely on a specific, overactive, or mutated protein (often an enzyme or receptor) to survive. Targeted therapies are engineered to bind directly to these proteins, shutting down their activity [190].

Next-generation tyrosine kinase inhibitors (TKIs) are engineered to counteract resistance mutations. In Chronic Myeloid Leukemia (CML), the tumor cells are driven almost entirely by the BCR-ABL fusion protein, a hyperactive tyrosine kinase. The Imatinib (Gleevec) works by slotting into the ATP-binding site of the BCR-ABL protein, effectively inhibiting its activity. This inhibition directly halts the signaling cascade that tells the cancer cells to divide uncontrollably. In the context of emerging resistance associated with new mutations in the BCR-ABL gene, particularly the T315I mutation, TKIs, such as ponatinib, are recognized for their efficacy [190]. These agents have demonstrated the ability to bind effectively to the mutated protein, thereby providing a viable treatment option. Similarly, in non-small cell lung cancer NSCLC with EGFR mutations, drugs like Erlotinib (Tarceva) block the EGFR receptor [190]. Osimertinib targets EGFR T790M by covalently binding to the C797 site in NSCLC [139] while brigatinib is effective against ALK G1202R mutations [140]. Agents like KRAS G12C inhibitors (e.g., sotorasib) address a breakthrough in targeting previously intractable KRAS mutations, showing promising activity in clinical trials [191]. In some cases, synthetic lethality occurs when the disruption of either of two genes alone is tolerable for a cell, but the disruption of both simultaneously causes cell death. Targeted therapies can be strategically employed to specifically inhibit the alternative pathway that cancer cells utilize for survival and proliferation. Normal cells have multiple pathways to repair DNA damage, ensuring their survival and proper function. Homologous recombination (HR), involving BRCA1 and BRCA2 genes, is the primary defense against genomic injury. Base excision repair (BER) pathway, involving PARP enzyme, is the other important mechanism [192]. Cancers with BRCA mutations already have a defective HR repair pathway, but are surviving by heavily relying on the PARP-mediated BER pathway. PARP inhibitors (olaparib, niraparib) work by blocking this critical backup repair system. When a PARP-inhibited cancer cell with a BRCA mutation experiences routine DNA damage, it cannot repair it because both repair pathways (HR and BER) are compromised. This leads to the accumulation of catastrophic DNA damage and selective cancer cell death, while sparing healthy cells that still have a functional HR pathway [190,192].

Cancer cells are adept at finding compensatory mechanisms. When one pathway is blocked, they often activate a parallel or downstream pathway to continue growing. So, using combination therapies that simultaneously target multiple nodes in a signaling network or entirely different pathways prevents the cancer from easily escaping treatment [193]. In HER2-positive breast cancer, combining trastuzumab with PI3K inhibitors helps counter resistance due to PTEN loss. Similarly, in MET-amplified tumors, crizotinib combined with EGFR-TKIs restores sensitivity by blocking alternative survival signals. In lung cancer, combining chemotherapy with EGFR inhibitors has been established to enhance therapeutic efficiency [139].

Additionally, during BRAF and MEK inhibition in melanoma, cancers with a BRAF V600E mutation use the MAPK/ERK pathway (often likened to a linear chain: BRAF-MEK-ERK) to drive growth. Relying solely on a BRAF inhibitor, such as dabrafenib, typically results in resistance because cancer cells often compensate by hyperactivating MEK downstream. Thus, by combining a BRAF inhibitor with a MEK inhibitor (trametinib), the entire pathway is blocked more effectively, leading to deeper and more durable responses. Another alternative approach involves the use of tyrosine kinase inhibitors (TKIs) in conjunction with immunotherapy. In this strategy, TKIs may specifically target intrinsic growth signals within cancer cells, while immunotherapeutic agents, such as checkpoint inhibitors (pembrolizumab), facilitate the activation of the immune system by removing its regulatory restraints. This combination attacks cancer on two fronts, directly killing cells and helping the immune system recognize and target them, thereby overcoming resistance mechanisms related to immune evasion [190].

TME-target modulation is another strategy affecting cancer cell behavior and manipulating chemotherapeutic resistance. PEGylated hyaluronidase (PEGPH20) breaks down hyaluronan in pancreatic tumors, enhancing gemcitabine access [132]. Anti-CD47 antibodies reprogram macrophages to clear tumor cells [194] and VEGF inhibitors improve immune infiltration by normalizing vasculature [132].

Inhibitors targeting ABC transporters can enhance drug efficacy by blocking these transporters from pumping drugs out of cells, a mechanism of multidrug resistance. Newer generation inhibitors, which specifically target transporters like ABCB1 and ABCC1, have shown promise in clinical trials by enhancing drug accumulation within tumor cells and blocking efflux pathways [174].

Epigenetic modifications, such as DNA methylation, also contribute to chemotherapy resistance. Drugs like azacytidine, guadecitabine, and decitabine reverse DNA methylation, reactivating tumor suppressor genes. Combination therapy of a drug with HDAC inhibitors enhances gene reactivation by restoring E-cadherin expression in breast cancer, leading to improved treatment outcomes [195].

Smart delivery systems, including antibody-drug conjugates (ADCs), liposomes, and nanoparticles, enhance tumor targeting and reduce systemic toxicity. Nanoparticles can release drugs against tumor-specific stimuli like pH or enzymes, improving drug accumulation at the tumor site and overcoming resistance attributed to poor drug penetration. Some targeted therapies work by directing the body’s own immune system to specifically destroy cancer cells, a mechanism completely different from chemical inhibition. These therapies flag cancer cells for destruction or empower immune cells to recognize and kill them.

Trastuzumab (Herceptin) specifically targets the HER2 protein present on breast cancer cells. In the mechanism of antibody-dependent cellular cytotoxicity (ADCC), this binding not only blocks growth signals but also flags the cancer cell, making it a clear target for immune cells, including Natural Killer (NK) cells [190].

Innovative therapies such as bispecific antibodies can target multiple mutations simultaneously, minimizing the risk of resistance escape. PROTACs (proteolysis-targeting chimeras) highlight a novel strategy of denaturing key oncogenic proteins rather than inhibiting, leading to overcoming resistance caused by protein overexpression [196].

Personalized treatment based on molecular profiling is driven by the mechanism of identifying specific resistance mechanisms in individual patients and customizing a treatment regimen according to the nature of the cancer. Liquid biopsy, biomarker-driven therapies, and adaptive treatment strategies are used to monitor disease progression and adjust therapies in real-time, ensuring that cancer treatments remain effective as the tumor evolves [132]. In liquid biopsy, by analyzing circulating tumor DNA (ctDNA) from a blood draw, doctors can non-invasively monitor for the emergence of new resistance mutations during treatment. This approach facilitates a proactive transition to a more effective treatment regimen prior to the clinical progression of the tumor. Furthermore, prior to treatment, biomarker-driven therapies involve the genetic profiling of tumors to identify specific targetable mutations (EGFR, ALK, BRCA). This approach ensures the appropriate medication is administered to the correct patient from the outset. In certain cancer types, such as hormone receptor-positive breast cancer, the implementation of adaptive therapy, which includes strategically planned breaks from treatment, can mitigate the selective pressure that permits fully resistant clones to prevail. Consequently, this strategy can extend the efficacy of the pharmacological agents involved [190].

## 6 Role of AI in Combating Resistance

The application of Artificial Intelligence (AI) to address drug target resistance has introduced innovative strategies in cancer treatment. It has emerged as a powerful tool that leverages machine learning (ML), deep learning (DL), and advanced mathematical algorithms to effectively interpret complex, multi-dimensional datasets. AI models for predicting drug response can either directly predict resistance or sensitivity outcomes or indirectly predict resistance by identifying biomarkers [197]. During direct prediction of drug response, AI models integrate multi-omics data to directly forecast whether a tumor will respond to a specific drug or not. Models based on Cell Line Data use the large public databases like Genomics of Drug Sensitivity in Cancer (GDSC) and Cancer Cell Line Encyclopedia (CCLE). Advanced models often use ensemble or deep learning methods for superior performance. DualGCN is a graph convolutional network that reports integration of drug structures and cancer omics data to predict treatment response more accurately than existing methods. This model analyzed more than 200 drugs [198]. Amongst all, CAY10603, a highly selective HDAC6 inhibitor, achieved the highest drug-wise Pearson correlation between the true and predicted IC_50_ values. This model most accurately captured how different cancer cell lines respond to CAY10603 [198]. This study highlights the promise of computational precision oncology in helping prioritize drugs that most likely work for individual patients based on tumor omics profiles.

The Morgan fingerprint, a digital representation of a drug’s chemical structure, investigates the chemical similarities between various drugs [199]. Examination of the Drug-Gene Network (DG-Net) and biological pathways offers a critical perspective on how the target genes may contribute to observed drug sensitivities. The PathDSP model helps predict a cancer cell’s sensitivity to a particular drug, measured by IC_50_, which indicates the concentration required to inhibit growth by 50%. This computational approach demonstrates promise for drug development and for guiding individualized medicine. However, there is a gap to clinical application, because the model was trained on cell lines, not patient tumors.

*In-silico* prediction of drug response in pre-clinical studies has also been underscored previously [200] in studies on kernelized multi-task learning (KMTL) method to predict drug sensitivity across cancer cell lines. This computational approach for predicting anti-cancer drug response has been reported as a strong and effective treatment strategy for drug development. Furthermore, Ensemble Methods with models like k-means, ensemble SVR and stacking methods (ELDAP) combine multiple algorithms (Elastic Net, SVM, Neural Networks) to boost prediction accuracy and robustness of the treatment.

Another category of AI models based on clinical data holds direct clinical relevance but is often constrained by smaller, single-cancer datasets. Multi-omics Integration model explored by Yi et al. combined radiomic features from CT scans, clinicopathological data, and SNP information to predict platinum resistance in ovarian cancer with high accuracy [201]. Johannet et al. developed a deep learning model, a Convolutional Neural Network (CNN), to analyze whole-slide images (WSI) of melanoma. This novel approach predicts how patients with advanced melanoma will respond to immune checkpoint inhibitors (ICIs), addressing the limitations of PD-L1 testing [202]. However, it is imperative to perform external validation across independent cohorts, rather than focusing solely on a specific cohort within these studies. It is pivotal to address this limitation in future research endeavors.

AI demonstrates exceptional ability in identifying molecular features linked to resistance, offering valuable insights into the underlying mechanisms. Transporters, an AI model designed to predict substrates of ATP-binding cassette (ABC) transporters such as P-glycoprotein, MRP1, and BCRP, play a crucial role in multidrug resistance [197]. Techniques like Genetic Algorithm–Correlation-Guided Support Vector Machine (GA-CG-SVM) and Consensus Self-Organizing Maps (CSOM) are effective in pinpointing compounds that are likely to be effluxed by these transporters. However, transferability and ethical considerations represent critical shortcomings inherent in this model, necessitating further research and development to address these issues effectively.

Artificial intelligence also conducts analyses of proteomic and metabolomic biomarkers, utilizing mass spectrometry data to identify protein or metabolite signatures associated with resistance. Machine learning techniques (Random Forests and SVM), can analyze transcriptomic data to identify gene expression signatures [197]. These signatures may help predict resistance to specific treatments, including oxaliplatin-based chemotherapy in colorectal cancer and temozolomide in glioblastoma.

AI goes beyond mere prediction and plays a transformative role in drug discovery by forecasting compound-protein interactions (CPIs). This can help identify new targets to overcome resistance. Several tools operate based on the principle of structure-based prediction. AlphaFold3 accurately predicts the 3D structures of biomolecular complexes [203]. This model elucidates the interactions between drugs and their respective protein targets and examines how mutations can induce resistance by altering binding sites. It demonstrates superior performance relative to specialized tools across several categories, including protein–ligand interactions, protein–nucleic acid interactions, and antibody–antigen interactions.

Geometric Deep Learning models such as SurfDock and BEACON leverage protein surface information to predict protein-ligand binding conformations with impressive accuracy. This advancement significantly aids in the virtual screening of new compounds that can potentially bypass resistance mechanisms [197]. These models possess the potential to revolutionize drug design by their integration with genomic, transcriptomic, and proteomic data.

## 7 Future Perspective

The future of oncological therapies lies in the integration of conventional and advanced therapeutic strategies for developing effective and personalized treatment regimens in patient care. The ongoing research has delivered exceptional breakthroughs in this direction. Consistent research [91,94] in the area of next-generation immunotherapies: checkpoint inhibitors, CAR-T cells, bispecific antibodies, and cancer vaccines will open new avenues for targeted cancer therapies. These approaches are patient-specific and will produce significant results in disease management. The future of oncology is increasingly oriented towards the development of therapeutics that target epigenetic regulators and employ RNA-based modalities for the treatment of challenging cancers. A comprehensive understanding of the molecular mechanisms underlying disease initiation and progression is essential. Consequently, innovative anticancer strategies may be developed based on these advanced therapeutic approaches. It is imperative to comprehend the mechanisms of resistance associated with both conventional and targeted therapies in order to achieve optimal outcomes in cancer management. Polytherapies targeting multiple resistance mechanisms and combining immunotherapy with other modalities offer more durable responses. Advanced molecular profiling and single-cell analyses will unravel tumor heterogeneity and microenvironment dynamics. CRISPR-based functional genomics is identifying resistance mutations, enabling tailored second-line strategies like MEK/SHP2 inhibitors for KRAS G12C tumors with NF1 [86,87]. Liquid biopsies using ctDNA provide real-time monitoring of resistance, detecting mutations like EGFR T790M months before progression [139]. Microfluidic platforms now isolate resistant cells for *ex vivo* testing [132]. Adaptive trial designs, such as basket and platform trials, match therapies to molecular profiles, accelerating validation of resistance-targeting drugs [140]. Continual research in these areas is essential for the future, as the challenge of resistance will be effectively addressed, leading to the development of more targeted therapies. Artificial intelligence integrates multi-omics and clinical data to predict resistance patterns and guide therapy selection. Recent advancements in artificial intelligence research has significantly enhanced the ability to predict resistance mechanisms and the scope of development of novel oncological therapies in the coming years [197,202]. These advancements signify a transition towards proactive and personalized cancer care, instilling optimism for the development of more effective treatment options.

## 8 Conclusion

The significance of targeted therapy in the realm of cancer management is profound. It hinges on a deep understanding of molecular pathology, allowing researchers to unveil the intricate mechanisms underlying cancer development. Scientists are relentlessly investigating new therapeutic targets, paving the way for more precise and effective treatment options. The traditional drug targets have positively impacted innumerable patients in controlling tumor progression, reducing the severity of symptoms, and providing palliative care. These strategies have mitigated patient distress over the years. However, with time, previously validated treatment regimens experience failure, and traditional approaches become redundant due to the development of resistance mechanisms. This prompted researchers to identify novel drug targets, elucidate the alternative pathways utilized by cancer cells to evade treatment, and ascertain the specific requirements of individual patients to develop tailored therapeutic interventions. Novel targets like KRAS G12C, CLDN18.2, TROP2, and epigenetic regulators have yielded positive results in cancer treatment. There is a huge scope to study new therapeutic targets and their pharmacological responses, corroborated by clinical trials. Currently, multiple clinical trials are underway aimed at evaluating the efficacy of these treatments and assessing any potential severe side effects associated with them. Collaborative efforts between researchers and health professionals are essential to accelerate the translation of scientific discoveries into effective cancer treatments. Continued advances in molecular biology, supported by AI-assisted data interpretation, will be pivotal in overcoming current barriers in drug resistance and therapeutic optimization.

## Data Availability

Not applicable.

## Other Declaration

The authors have used Grammarly to ensure that the English text is grammatically correct during the writing process. The manuscript was subsequently reviewed and edited for language. The authors take full responsibility for the content of the publication.

## Abbreviations

EGFR: Epidermal Growth Factor Receptor
HER: Human Epidermal Growth Factor Receptor
ER: Estrogen Receptor
AR: Androgen Receptor
VEGF: Vascular Endothelial Growth Factor
PARP: poly ADP-ribose polymerase
ADCC: Antibody-Dependent Cellular Cytotoxicity
NTRK: Neurotrophic Tyrosine Receptor Kinase
ARSIs: Androgen Receptor Signaling Inhibitors
c-MET: Mesenchymal-Epithelial Transition Factor
hTOP: Human Topoisomerase
KRAS: Kirsten Rat Sarcoma Viral Oncogene Homolog
G12C: Glycine-to-Cysteine substitution at codon 12
RTK: Receptor Tyrosine Kinase
SHC: Src Homology 2 Domain Containing Transforming Protein
GRB2: Growth Factor Receptor-Bound Protein 2
SOS: Son of Sevenless (a guanine nucleotide exchange factor)
GAP: GTPase-Activating Protein
GDP: Guanosine Diphosphate
GTP: Guanosine Triphosphate
RAS: Rat Sarcoma Protein
RAF: Rapidly Accelerated Fibrosarcoma Kinase
MEK: Mitogen-Activated Protein Kinase Kinase (MAP2K)
AKT: Protein Kinase B (also known as PKB)
TROP2: Trophoblast Cell-Surface Antigen 2
IGF-2: Insulin-like Growth Factor 2
mAb: Monoclonal Antibody
JAK/STAT: Janus Kinase/Signal Transducer and Activator of Transcription
ERK: Extracellular Signal-Regulated Kinase
PI3K/AKT: Phosphoinositide 3-Kinase/Protein Kinase B
IP3: Inositol 1,4,5-trisphosphate
TIM-3: T-cell Immunoglobulin and Mucin-domain containing-3
LAG-3: Lymphocyte-activation gene 3
FGL1: Fibrinogen-like protein 1
MHC: Major Histocompatibility Complex
Th1: T helper type 1
AI: Artificial Intelligence
ML: Machine learning
DL: Deep Learning
DualGCN: Dual Graph Convolutional Network
PathDSP: Pathway-Based Drug Sensitivity Prediction
ELDAP: Ensemble Learning for Drug Activity Prediction
GA-CG-SVM: Genetic Algorithm–Correlation-Guided Support Vector Machine
